# Functionalization of Saturated and Unsaturated Hydrocarbons with Electrophilic Anions [B_12_X_11_]^−^ (X = Br, I) in the Gas Phase and at Interfaces

**DOI:** 10.1021/acs.jpca.5c08384

**Published:** 2026-01-30

**Authors:** Markus Rohdenburg, Jaskiran Kaur, Ashley J. Galligan, Aby-Paul Benny, Kay-Antonio Behrend, Xin Ma, Stanislav Petrovskii, Kirill Monakhov, Hilkka I. Kenttämaa, Jonas Warneke

**Affiliations:** † Wilhelm-Ostwald-Institut für Physikalische Und Theoretische Chemie, 9180 Universität Leipzig, Linnéstr. 2, Leipzig 04103, Germany; ‡ James Tarpo Jr. and Margaret Tarpo Department of Chemistry, 311308 Purdue University, 560 Oval Drive, West Lafayette, Indiana 47907, United States; § Department of Chemistry, 2358 University of Virginia, 409 McCormick Rd, Charlottesville, Virginia 22904, United States; ∥ Leibniz Institute of Surface Engineering (IOM), Permoserstr. 15, Leipzig 04318, Germany

## Abstract

Electrophilic dodecaborate fragment ions [B_12_X_11_]^−^ are among the most reactive anions known and are capable of converting inert reagents to charged compounds through covalent bonding. In this study, we investigate the reactivity of [B_12_X_11_]^−^ (X = Br, I) with a series of hydrocarbons in two environments: (1) in the gas phase, using ion-molecule reactions and collision-induced dissociation (CID), and (2) at vacuum/solid interfaces, via fragment ion deposition onto surface layers of reagents. In the gas phase, *n-*alkyl groups of hydrocarbons are bound to [B_12_X_11_]^−^ via the substitution of a proton by the electrophilic vacant boron atom. However, hydrocarbons with double and triple bonds are bound directly to [B_12_X_11_]^−^, resulting in strongly bound adducts with characteristic fragmentation behavior. Aromatic compounds can bind to the vacant boron atom in [B_12_X_11_]^−^ by donating electron density from the aromatic unit, forming a quasi-tetrahedrally coordinated carbon atom, and the adduct fragments upon CID to regenerate [B_12_X_11_]^−^ ions. Contrary to the gas-phase results, [B_12_X_11_]^−^ reacts with all hydrocarbons at layer interfaces via proton substitution, regardless of the degree of saturation. Computational investigations rationalize that the thermochemically most favorable geometry of the [B_12_X_11_]^−^ —hydrocarbon adduct can often explain their observed fragmentation behavior in the gas phase. However, in the condensed phase, low-lying transition states allow rearrangement into the proton substitution binding mode, which is favorable due to entropic effects (e.g., proton dissipation in the layer). These results emphasize the possible differences in the formation of products of reactive ions in the gas phase and at interfaces, contributing to the selectivity control in reactive ion deposition experiments and providing a foundation to apply the “universal binder” [B_12_X_11_]^−^ in analytical and preparative mass spectrometry for charge tagging of nonpolar organic molecules.

## Graphical abstract

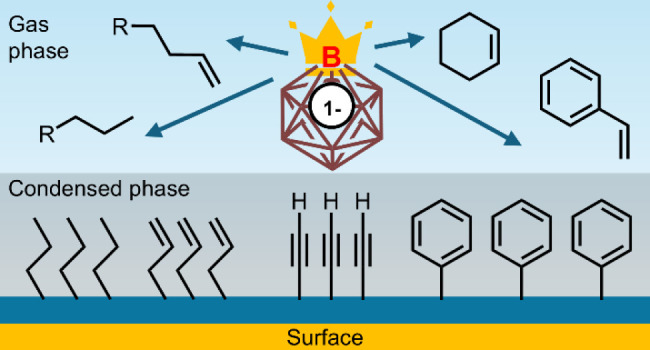

## 1 Introduction

Dodecaborate fragment ions [B_12_X_11_]^−^ (X = halogen, CN) are the most reactive molecular anions known.^1,2^ These ions are generated upon collision-induced dissociation (CID) of their highly stable precursor ions, the *closo*-dodecaborate dianions [B_12_X_12_]^2–^. The vacant boron atom in [B_12_X_11_]^−^ is partially positively charged and reacts as a strong Lewis acid/electrophile, despite the overall negative charge of the ion. The exceptionally high reactivity of [B_12_X_11_]^−^ makes it a “universal ionic binder” that converts inert neutral compounds to molecular anions by covalent bonding. Reagents with groups that are Lewis bases (L)—including very weak bases such as N_2_ and noble gases^1–4^ —are bound directly to the electrophilic vacant boron atom via the lone pair of L (reaction 1). If no basic groups are available, e.g., in the case of alkanes, σ-bonds can be heterolytically cleaved, and the anionic site formed in the alkane binds to the electrophilic vacant boron atom (reaction 2).^5,6^ The cation generated by heterolytic cleavage (e.g., H^+^ in the case of alkanes) remains electrostatically bound to the resulting dianion in gas-phase reactions. The generation of a covalently bound dianion following the reaction of a singly charged [B_12_X_11_]^−^ ion with a neutral reagent can only be explained by the counterintuitive strong electrophilic/Lewis acid character of the vacant boron atom in these anions.

1 $${[B_{12}X_{11}]}^{-}+L\to {[B_{12}X_{11}-L]}^{-}$$

2 $${[B_{12}X_{11}]}^{-}+Y-Z\to {[Z]}^{+}{[B_{12}X_{11}-Y]}^{2-}$$

We have developed methods to functionalize complex neutral molecules on surfaces with [B_12_X_11_]^−^ generated in the gas phase by using fragment ion deposition.^7,8^ Converting the surface-adsorbed molecules into covalently charge-tagged compounds enables sensitive analysis using liquid extraction surface analysis mass spectrometry (LESA-MS) in negative ion mode.^9,10^ These charge-tagged molecules often exhibit altered fragmentation behavior compared to the nonmodified molecules, thus yielding complementary structural information. If highly reactive [B_12_X_11_]^−^ in a low-pressure gas phase reaches a layer interface, reactions occur “on contact,” and highly selective binding to interfacial active groups is observed.^5,9,10^ Therefore, identifying the molecular sites to which [B_12_X_11_]^−^ attaches reveals the interfacial activity and orientation of molecules within the layer. Previous studies have demonstrated that [B_12_X_11_]^−^ binds to alkyl groups of complex organic molecules on surfaces,^5,9,10^ although binding to polar functional groups is thermochemically favored. This can be rationalized by the high interfacial activity of the nonpolar alkyl chains at the vacuum interface. A fundamental mechanistic understanding of the reactions between [B_12_X_11_]^−^ and alkyl groups is of paramount importance for the rational functionalization of molecules at interfaces with anionic dodecaborate charge tags. Such reactions will open new avenues for the generation of new dodecaborate conjugate prototypes with potential applications in therapeutics,^11–14^ materials science, e.g., for nonlinear optics and as special anionic surfactants,^15–21^ and analytics.^9,22^

In previous studies, reaction mechanisms of [B_12_X_11_]^−^ with different alkanes were investigated in the gas phase, and it was shown that binding occurs via reaction 2 by cleaving either a C–H or a C–C bond.^5,6^ The CID mass spectra of the adducts [B_12_X_11_(C_6_H_14_)]^−^ with all five hexane isomers studied differ depending on the alkane structure. In contrast, the CID mass spectra of adducts with the constitutional isomers hexene and cyclohexane [B_12_X_11_(C_6_H_12_)]^−^ did not show any differences. Hexenes bind to the free boron atom via the electron-rich C=C double bond (reaction 1) resulting in a structure that is best described as [B_11_X_11_B]^2–^–CH_2_–CH^+^–C_4_H_9_.^6^ Bound hexenes isomerize efficiently, as indicated by almost equal CID mass spectra independent of the alkene structure, showing the exclusive formation of [B_12_X_11_(C_2_H_4_)]^−^. Isomerization has been attributed to the carbocation center present in the product. In the case of cyclohexane, gas-phase infrared spectroscopy showed that reactions with [B_12_X_11_]^−^ open the six-membered ring of the reagent, generating the same structure as observed for hexene binding.^6^

In the case of ion deposition experiments using [B_12_X_11_]^−^, bond formation with terminal methyl groups of alkyl chains has been observed, and the same mechanism as determined for the gas-phase reactions has been proposed (see Scheme 1). The direct binding of [B_12_X_11_]^−^ to C=C bonds in reagents at interfaces has not been investigated thus far. Since the gas-phase product of [B_12_X_11_]^−^ with alkenes contains a carbocation, it is unlikely that the equivalent product could exist in the condensed phase for extended times without further reactions. Substitution of a proton with the electrophilic [B_12_X_11_]^−^ ion (equivalent for the case of *n*-alkanes) was observed when [B_12_X_11_]^−^ ions reacted with phenyl groups at molecular layer interfaces and within bulk layers.^9,23^ However, in this case, no gas-phase data are available to reveal the reaction of isolated [B_12_X_11_]^−^ with aromatic units without the influence of the condensed phase/interface. Scheme 1 depicts the current availability of gas-phase and ion deposition data (green checkmarks) for the reactions of [B_12_X_11_]^−^ with different hydrocarbon structural elements. In this study, we investigated the reactions marked with red question marks in Scheme 1, which were not reported previously. DFT investigations were utilized to rationalize hydrocarbon-structure-dependent reaction patterns. Our results show that the binding modes operating in the gas phase are, in general, not predictive of binding modes operational at the layer interface.

**Scheme 1:**
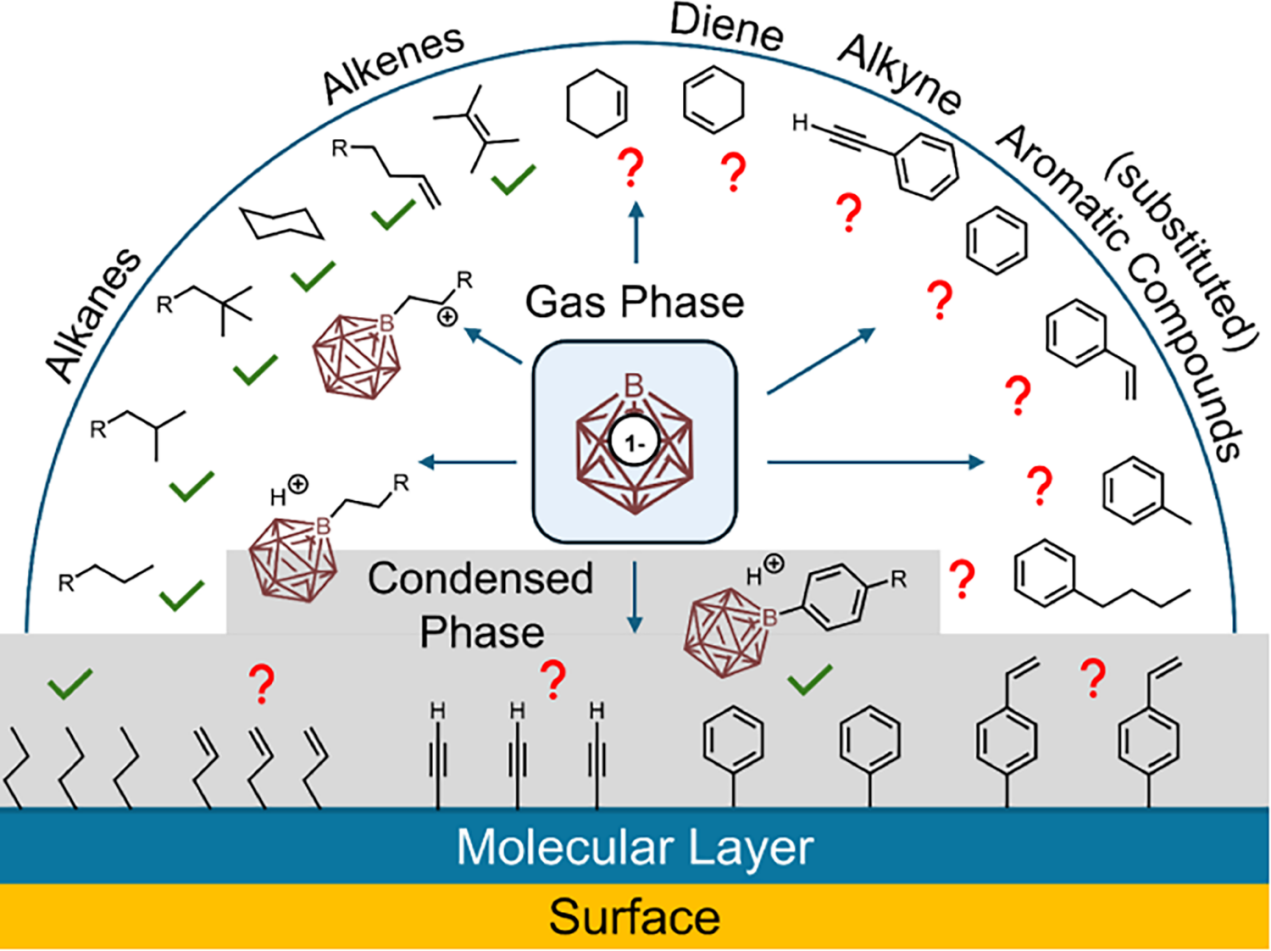
Overview of the Experiments Performed Previously (Green Checkmarks) and within This Study (Red Question Marks) in order to Complement the Understanding of Binding of [B_12_X_11_]^−^ Ions to Hydrocarbons with Different Structural Elements (*n*-Alkyl, Branched, Double Bond, Triple Bond, Aryl, and Combinations Thereof) Both in the Gas Phase and Condensed Phase^a^

a Several identical structural elements next to each other symbolize a layer boundary between these structures and the vacuum. Note that, for the sake of visual clarity, the halogen substituents of [B_12_X_11_]^−^ were omitted. Here and in the following, the use of brown color for the B_12_ scaffold indicates [B_12_Br_11_]^−^ and purple indicates [B_12_I_11_]^−^. The type of bond formation observed is independent of the identity of X.^5^

Reactive landing of ions has been studied extensively in the past.^24–32^ New developments in preparative mass spectrometry employing electrospray ionization (ESI) sources and facilitating deposition with high ion currents^8,33–35^ have enabled the synthesis of macroscopic layers on surfaces by using mass-selected ions and their fragments generated in the gas phase. Therefore, ionic reactive intermediates observed in mass spectra have the potential to become building blocks for technologically relevant thin layer synthesis.^7^ However, controlling the bond formation of highly reactive ions is challenging. Herein, we aim to contribute to conceptual developments in selective reactions of fragment ions at interfaces by complementing the knowledge about reactions of the universal binder [B_12_X_11_]^−^ with hydrocarbon residues, both in the gas phase and at vacuum/solid interfaces.

## 2 Experimental Methods

### 2.1 Chemicals

K_2_[B_12_Br_12_] and K_2_[B_12_I_12_] were obtained from the Jenne group (University of Wuppertal, Germany), where the salts were synthesized according to previously published procedures.^36,37^

The hydrocarbons *n*-heptane, 2,3-dimethylbutane, 1-hexene, 2,3-dimethylbut-2-ene, cyclohexane, methylcyclohexane, cyclohexene, cyclohexa-1,3-diene, benzene, toluene, ethylbenzene, styrene, and phenylacetylene were purchased from Sigma-Aldrich; ethylcyclopentane was purchased from Tokyo Chemical Industry America; *n*-pentylbenzene was purchased from Thermo Fisher Scientific; and 1-propenylbenzene was purchased from Oakwood Chemical. All hydrocarbons used in the gas-phase ion-molecule reactions (see the structures in Figure 1) were used without further purification.

**Figure 1:**
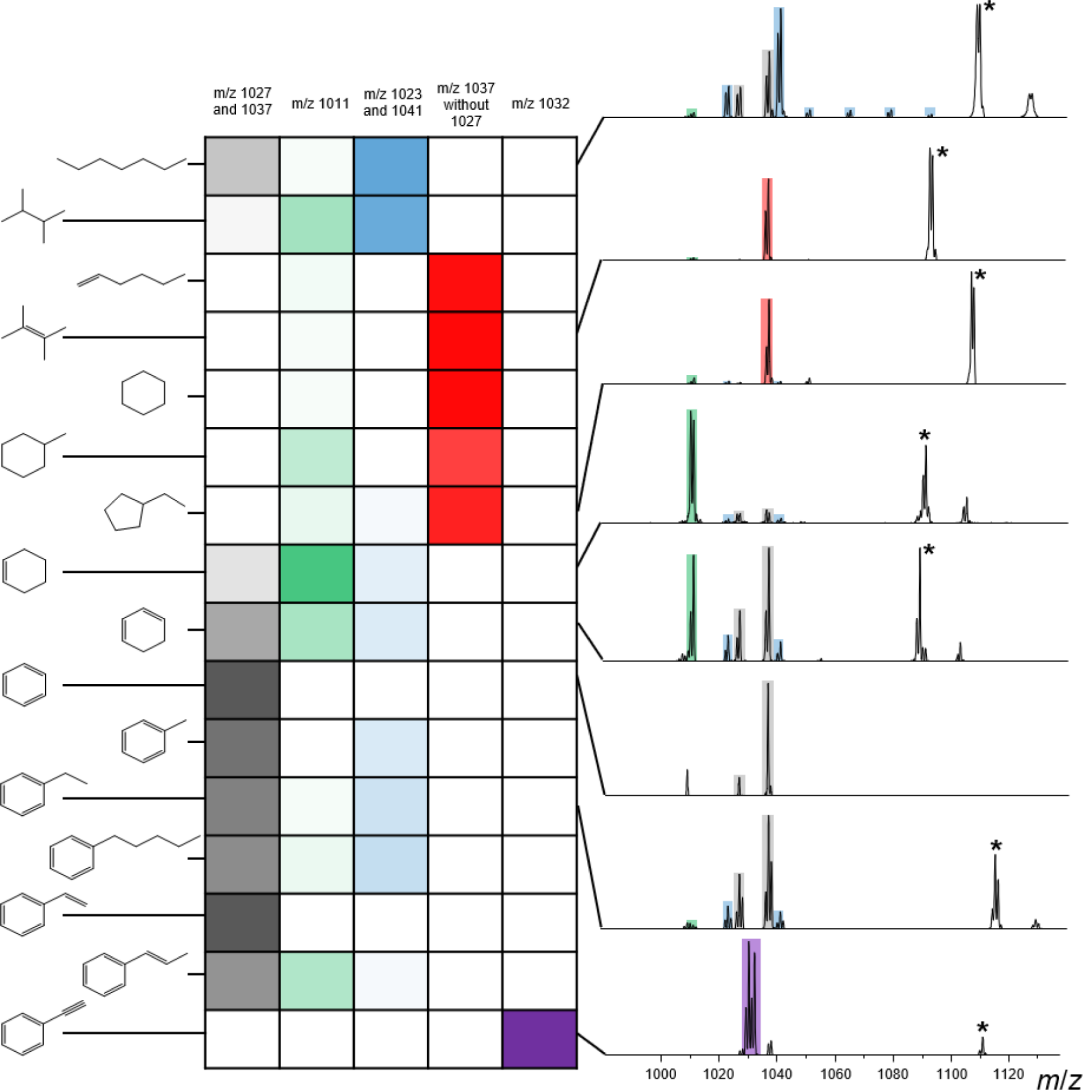
Fragment ions observed upon CID of the gas-phase adducts of [B_12_Br_11_]^−^ with various hydrocarbons containing different degrees of branching and saturation (aliphatic, olefinic, acetylenic, cyclic, aromatic; see left side). The table indicates the relative abundances of characteristic fragment ions resulting from the CID of the [B_12_Br_11_-hydrocarbon]^−^ adduct as a color code (darker = more abundant). Selected corresponding fragment ion mass spectra, acquired after isolation and fragmentation of these adducts, are shown on the right side. Remaining signals of the precursor ions are marked with asterisks. Colored bands represent respective signals from the table. The fragment ion mass spectra not shown here can be found in the Supporting Information, Figure S15.

The following substances, used for codeposition in the fragment ion deposition experiments, were purchased from Sigma-Aldrich and used without further purification: (i) 1-pyrenesulfonic acid sodium salt (>97.0%), (ii) vinylphosphonic acid (97%), and (iii) sodium 4-vinylbenzenesulfonate (>90%).

4-(4-(Ethynyl-d)­phenyl)-1-methylpyridinium trifluoromethanesulfonate was used to obtain ion (iv) (see Figure 2a) in +ESI (electrospray ionization) experiments and synthesized according to the description in the Supporting Information (Scheme S1). NMR spectra can be found in the Supporting Information, Figure S1–S7.

**Figure 2:**
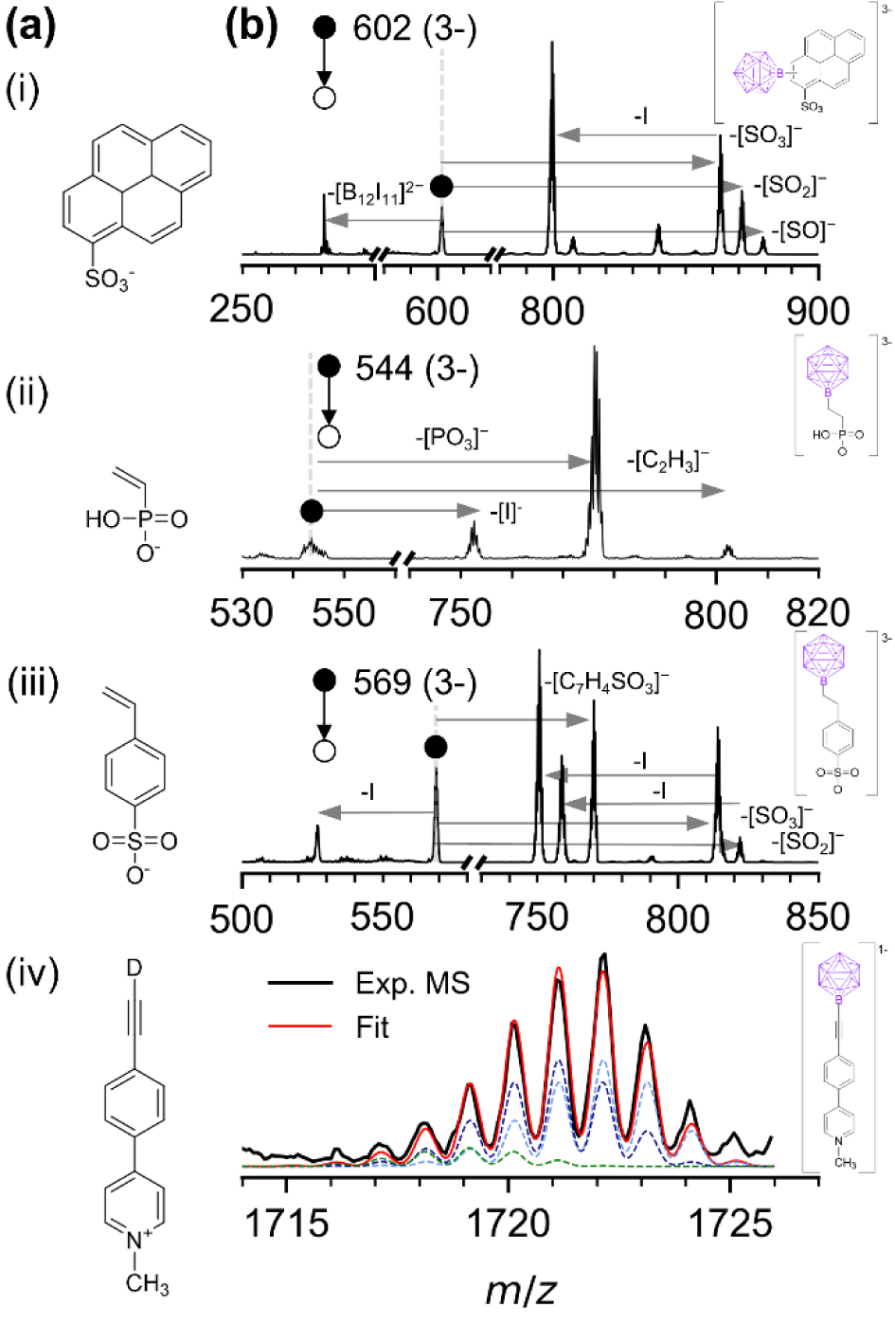
(a) Formulas of ions codeposited with [B_12_I_11_]^−^ in sequential ion soft-landing experiments: (i) pyrene sulfonate, (ii) vinyl phosphonate, (iii) 4-vinylstyrenesulfonate, (iv) deuterated *N*-methyl-4-(4-ethynylphenyl-1-yl)­pyridinium. (b) Corresponding MS^2^ (for i–iii) and MS (for iv) spectra, confirming adduct formation with [B_12_I_11_]^−^. The proposed structure of the main adduct isomer is shown on the right of the spectra. For (iv), the MS in the *m*/*z* region of the adduct was fitted to a combination of simulated isotopic patterns of the adduct (green) and ion pairs of cation (iv) (light blue) and its fully protonated form (dark blue) with the major side product of the reaction, [B_12_I_11_H]^2–^. The sum of all fitted components is displayed in red. Only components with a contribution of >1% to the overall sum fit (red) are displayed.

### 2.2 Gas-Phase Ion-Molecule Reactions and Collision-Induced Dissociation (CID)

Gas-phase ion-molecule reactions and product analyses were conducted using a modified Thermo Scientific LTQ XL linear quadrupole ion trap (LQIT) mass spectrometer equipped with an ESI source. The analyte solutions were prepared in methanol and were directly introduced into the ESI source at a flow rate of 10–15 μL/min by using a 500 μL Hamilton syringe. The ESI source conditions used were 3 kV spray voltage, 20–30 (vendor-specific arbitrary units) of sheath gas (N_2_), 10 (vendor-specific arbitrary units) of auxiliary gas (N_2_), and 275 °C capillary temperature. After transfer of the ionic analytes into the gas phase, the ions were transferred into the ion trap, isolated using an isolation width of 0.5–2.0 *m*/*z* units and a *q* value of 0.25 and were allowed to react with different reagents for up to 10,000 ms before all ions were ejected for detection. Reagents were introduced into the ion trap (containing 2 mTorr of helium buffer gas) via an external reagent mixing manifold.^38^ The reagents were introduced into the manifold by using a syringe pump at a flow rate of 1–2 μL/h and diluted with helium before entering the ion trap through a split valve. For CID experiments, the advanced scan features of the LTQ Tune Plus interface were used. The isolated ions were subjected to CID (collision energy of 5–30 arbitrary units) for 30 ms by using helium as the collision gas. All mass spectra acquired were an average of at least 15 individual mass spectra. Xcalibur version 4.1 was used to process the data.

### 2.3 Fragment Ion Deposition Experiments

Fragment ion deposition was performed using a previously described ion soft-landing instrument optimized for fragment ion deposition.^8,23^ In individual, sequential deposition experiments, 1 pmol of each of the organic ions containing different hydrocarbon moieties (alkyl, aryl, alkenyl, alkynyl; see structures in Figure 2a) was deposited on gold surfaces by mass-selective ion soft-landing, followed by deposition of 1 pmol of [B_12_I_11_]^−^ ions. This procedure was repeated multiple times in a sequential deposition experiment enabled by our dual-polarity ion soft-landing instrument with two independent ESI sources (total deposited amount was typically in the range of 15–50 pmol for each ion).^35^ This experimental procedure increased the number of interfaces between the codeposited species and led to enhanced adduct formation in previous studies on functionalization with reactive fragment ions.^9,39^ After deposition, the gold surfaces were removed from the instrument, and the deposited substance was dissolved and analyzed using LESA-MS. Further details on the deposition process can be found in the Supporting Information. Results from kinetic energy measurements of the deposited ions are shown in the Supporting Information, Figures S8–S13.

### 2.4 LESA-MS

A TriVersa NanoMate (Advion) for LESA^40^ was used for nano-ESI analysis. A small portion of the layer (1 to 2 mm^2^) was dissolved in 2 μL of 80:20 MeOH:H_2_O (v/v) and subjected to chip-based nano-ESI analysis. This procedure minimizes the required sample volume and is highly sensitive in comparison to previous ESI-MS analyses of soft-landed ions. Mass spectra were acquired on a Thermo Fisher LTQ XL Orbitrap spectrometer (Thermo Scientific GmbH, Bremen, Germany).

### 2.5 DFT Investigations

DFT calculations were performed using Gaussian 16, rev. C.02 program.^41^ Geometry optimization was carried out using the B3LYP-D3BJ/def2-TZVPP level of theory.^42–47^ Subsequent frequency calculations were performed to ensure that the obtained stationary points correspond to either minima or transition states on the potential energy surface (PES). For 0 K attachment enthalpies (Δ*H*_0K_), the Basis Set Superposition Error (BSSE) was corrected for in a counterpoise calculation on the optimized geometry.^48,49^ All outputs of relevant calculations are published in ioChem-BD and can be retrieved via the following link: 10.19061/iochem-bd-6-606

## 3 Results and Discussion

### 3.1 Gas-Phase Ion-Molecule Reactions of [B_12_X_11_]^−^ with Different Hydrocarbons

In the gas phase, [B_12_X_11_]^−^ ions reacted with all hydrocarbons (C_*n*_H_*m*_) shown in Scheme 1 by forming ions with the molecular formula [B_12_X_11_(C_*n*_H_*m*_)]^−^. The adducts were isolated using a narrow isolation width (Section 2.2) from the center of the broad isotopic distribution that results from the abundant ^10^B and ^11^B isotopes in [B_12_X_11_]^−^. The *m*/*z* values stated in the following text correspond to the signal of the most abundant isolated ion. CID of these adducts results in characteristic fragmentation patterns that provide an indication of the binding mode of the [B_12_X_11_]^−^ ion. The results are shown and discussed for the particular case of X = Br; see Figure 1. A color-coding system identifies characteristic fragments formed upon CID that indicate a specific binding position of [B_12_X_11_]^−^ at the hydrocarbon. The darker the color, the larger the percentage of the corresponding fragments out of all the fragment ions observed in Figure 1. Consistent with preliminary work,^5^ reactions using [B_12_I_11_]^−^ show similar results and are exemplarily provided in the Supporting Information, Figure S14.

*Gray*: Attaching the hydrocarbon via a H–C bond to the vacant boron atom of [B_12_Br_11_]^−^ without breaking the bond (forming a B–H–C structural element) results in the formation of an adduct that dissociates upon CID to regenerate the reactants, i.e., [B_12_Br_11_]^−^ (*m*/*z* 1009) and the intact hydrocarbon. However, [B_12_Br_11_]^−^ was only observed in low abundance in the fragment ion mass spectra due to its high reactivity. The vacant boron atom of [B_12_Br_11_]^−^ reacts with H_2_O and N_2_ in the residual gas atmosphere of the mass spectrometer, yielding [B_12_Br_11_(H_2_O)]^−^ (*m*/*z* 1027) and [B_12_Br_11_N_2_]^−^ (*m*/*z* 1037), respectively, in high abundance. Observation of these ions thus indicates that no covalent B–H or B–C bond had been formed as the adduct dissociates into the reactants ([B_12_Br_11_]^−^ and hydrocarbon). It should be noted that multiple assignments are possible for ions of *m*/*z* 1037, as the masses of N_2_ and C_2_H_4_ cannot be distinguished with the resolution of our instrument. To unambiguously assign an elemental composition to the ions of *m*/*z* 1037, its ratio to *m*/*z* 1027 (H_2_O adduct) must be taken into account. If the abundance ratio of the ions of *m*/*z* 1027 and 1037 is equal to that observed for the reaction products of [B_12_Br_11_]^−^ in the ion trap without introducing a hydrocarbon reagent, observation of *m*/*z* 1037 in the CID mass spectra of [B_12_X_11_(C_*n*_H_*m*_)]^−^ must be exclusively assigned to the N_2_ adduct. Otherwise, contributions from [B_12_Br_11_(C_2_H_4_)]^−^ must be considered, indicating another binding mode, *vide infra*. Figure 1 shows that dissociation of the [B_12_Br_11_]^−^ —hydrocarbon adduct into the reactants is only a minor CID pathway for alkanes, alkenes, and cyclic saturated alkanes. For ring structures containing double bonds and aromatic hydrocarbons, however, this reaction pathway is dominant, with the exception of the reactions with cyclohexene.

*Red*: Binding [B_12_Br_11_]^−^ to the double bond of an alkene or the cleavage of a C–C bond in an aliphatic ring system leads to the formation of [(B_12_Br_11_)^2–^–CH_2_–(CH)^+^–C_*n*_H_2*n*+1_]^−^ (see gas-phase adduct structure in Scheme 1).^6^ CID of such products generates almost exclusively the fragment ion [B_12_Br_11_(C_2_H_4_)]^−^ by elimination of the alkene. Therefore, an abundant ion of *m*/*z* 1037 (without simultaneous detection of the H_2_O adduct at *m*/*z* 1027) is characteristic of open-chain alkenes and aliphatic ring systems, with or without alkyl branches; see the comparison of 1-hexene, 2,3-dimethylbut-2-ene, cyclohexane, and ethylcyclopentane in Figure 1. This finding supports the previous conclusion that [B_12_Br_11_]^−^ opens aliphatic ring structures to alkenes, which then isomerize due to the carbocation character of the CH group in [(B_12_Br_11_)^2–^–CH_2_–(CH)^+^–C_*n*_H_2*n*+1_]^−^.^6^

*Blue*: Substituting a proton in hydrocarbons with [B_12_Br_11_]^−^ leads to the formation of [H]^+^[B_12_Br_11_–CH_2_–C_*n*_H_2*n*+1_]^2–^. The substituted proton remains bound to the dianion on the edge of two halogen substituents and can migrate across the surface of the dodecaborate ion (the transition state for proton movement between neighboring halogen–halogen edges is about 20 kJ/mol).^5^ CID of the product ion results in the characteristic formation of [B_12_Br_11_–CH_2_]^−^ (*m*/*z* 1023) and is observed when terminal methyl groups are present in the hydrocarbon. The highly electrophilic carbon atom of [B_12_Br_11_–CH_2_]^−^ binds to residual H_2_O forming [B_12_Br_11_–CH_2_–OH_2_]^−^ (*m*/*z* 1041). In the case of reagents with longer *n*-alkyl units, [B_12_Br_11_–(CH_2_)_*m*_]^−^ product ions with *m* ≥ 2 were also observed. The substitution of a proton is the dominant reaction for *n*-alkanes.

*Green:* Hydride (H^–^) abstraction by the electrophilic vacant boron atom leads to the formation of a [B_12_Br_11_H]^2–^[C_*n*_H_*m*‑1_]^+^ ion pair. CID results in the formation of the [B_12_Br_11_H]^2–^[H]^+^ ion (*m*/*z* 1011), which is formed by the elimination of an alkene. H^–^ abstraction is observed as the dominant reaction when a stable carbocation can be formed. This is the case for branched alkanes containing tertiary carbon atoms (C_tert_) as in 2,3-dimethylbutane, and allylic carbon atoms, as in cyclohexene and 1-propenylbenzene (see Figure 1).

*Purple:* Binding [B_12_Br_11_]^−^ to phenylacetylene leads to [B_12_Br_11_–HC≡C-Ph]^−^, which is strongly bound—presumably via an η_2_ bond—and does not dissociate. Likely, the conjugated π-system prevents fragmentation of the hydrocarbon, and instead, HBr is eliminated. Note that, in contrast to the other cases discussed, the fragment ion colored in purple is thus not generally diagnostic for all possible reactants containing a triple bond, since its precise *m*/*z* depends on the rest of the R group to which the triple bond is attached.

The results in Figure 1 show a clear correlation between the hydrocarbon structures and fragmentation of the [B_12_X_11_]^−^ adducts. We hypothesize that the structure-dependent dissociation is indicative of the binding motif. Particularly, [B_12_X_11_]^−^ binds to aryl compounds without cleaving a C–C or C–H bond, as the adduct dissociates to regenerate the [B_12_X_11_]^−^ ions. In the case of the alkyl-substituted aromatic compounds, fragmentation mass spectra indicate that the formed adduct is a mixture of constitutional isomers: both binding via the aryl units (without C–C or C–H bond cleavage) and proton substitution at the alkyl group (via covalent B–C bond formation) occur. The direct dissociation of the aryl-bound [B_12_Br_11_]^−^ adduct appears to be in direct contrast to results from fragment ion deposition experiments. It was shown that [B_12_X_11_]^−^ ions bind surface-active phenyl groups in layers of both the dipeptide phenylalanylproline (PhePro)^9^ and tetraphenylphosphonium ions ([PPh_4_]^+^) via proton substitution.^23^ The resulting B–C_phenyl_ bonds do not dissociate in the CID experiments. This difference motivated a comparative study of the binding of [B_12_X_11_]^−^ ions to different hydrocarbon structural elements at interfaces.

### 3.2 Fragment Ion Deposition of [B_12_X_11_]^−^ into Layers with Different Hydrocarbon Residues at the Vacuum/Solid Interfaces

The hydrocarbons investigated in the gas phase (Section 3.1) cannot be directly adsorbed in higher coverages on a surface at room temperature for binding experiments because they are too volatile and would evaporate under the high-vacuum conditions of the soft-landing instrument. To investigate reactions with compounds containing equivalent functional groups at a layer interface, molecular ions containing such functional groups were deposited on the surfaces using ion soft-landing.

Four organic ions (i–iv, see Figure 2a) were selected for interfacial binding experiments and separately deposited on gold surfaces. Assuming that these ions orient with their polar groups toward the ion layer/polarizable gold surface and their nonpolar groups toward the vacuum,^5,9,50^ aromatic units (i), double bonds (ii), styrene units (iii), and acetylene groups (iv) were available at the vacuum interface for reactions with [B_12_X_11_]^−^. In four independent experiments, the organic ions in Figure 2a and [B_12_I_11_]^−^ were sequentially deposited on gold surfaces (1 pmol each) until a total amount in the range of 15–50 pmol of each ion was deposited on a circular spot of 2–3 mm^2^. The grounding of the deposition surface leads to charge compensation at the interface. Revealing the details of charge-balancing reactions in deposited layers is a current topic of ion soft-landing research; in the case of anion deposition, H^+^ may balance the charge at the interface,^23^ and protonation of the deposited ions (i)–(iii) (Figure 2a) is expected. [B_12_X_11_]^−^ was found to react with all codeposited compounds (i–iv), independent of their polarity. LESA-MS of the generated surface layers revealed that products of the deposited anions (i)–(iii) can be detected by mass spectrometry as doubly and triply negatively charged ions. The latter ions are missing a proton compared with the mass-selected organic anion. The product ion with cation (iv) is detected as a singly charged anion and is also missing one proton compared with the two reactants. The charge state of the products demonstrates the substitution of H^+^ by [B_12_X_11_]^−^. We assume that the dianions detected for (i), (ii), and (iii) are formed due to protonation of the organic basic group during ESI analysis of the products. Note that we selected [B_12_X_11_]^−^ ions with X = I for these experiments to avoid confusing elimination of Br^–^ with the almost isobaric SO_3_^–^ in CID experiments. Near-isobaric eliminations are difficult to distinguish due to the very broad isotopic pattern of brominated dodecaborate anions. However, we ensured that the deposition of [B_12_Br_11_]^−^ yields products with similar fragmentation behavior, as indicated for two examples in the Supporting Information (Figure S16).

While proton substitution by [B_12_X_11_]^−^ with gaseous alkanes results in H^+^ bound to [B_12_X_11_–CH_2_–C_*n*_H_2*n*+1_]^2–^, such an abstracted proton can be easily separated from the weakly basic anion in surface layers or upon dissolving the substance for LESA analysis. CID of the adducts formed with anions i–iii (Figure 2b) shows the elimination of the polar groups of the organic moieties from these adducts and confirms that [B_12_X_11_]^−^ was bound to the hydrocarbon residues and not to the oxygen-containing functional groups. A summary of the observed fragmentation reactions is given in Scheme 2. In the case of B–O bond formation, [B_12_X_11_OH]^2–^ would be formed in MS^*n*^ experiments, as previously shown.^9,50^ However, [B_12_X_11_OH]^2–^ was only observed during fragmentation of the adducts of the small ion (ii), in particular during CID of the respective doubly charged ion [B_12_I_11_C_2_H_4_O_3_P]^2–^ (see Supporting Information, Figure S17). The formation of small amounts of O-bound adducts is also evidenced by the elimination of the full hydrocarbon residue (−[C_2_H_3_]^−^) from the triply charged adduct of ion (ii). We note that [B_12_X_11_OH]^2–^ formation was hardly observed for the larger ions and thus conclude that the small nonpolar carbon chain of ion (ii)—although constituting the major binding site—cannot shield the larger PO_3_ group completely, thus resulting in the formation of products with B–C and B–O bonds.

**Scheme 2:**
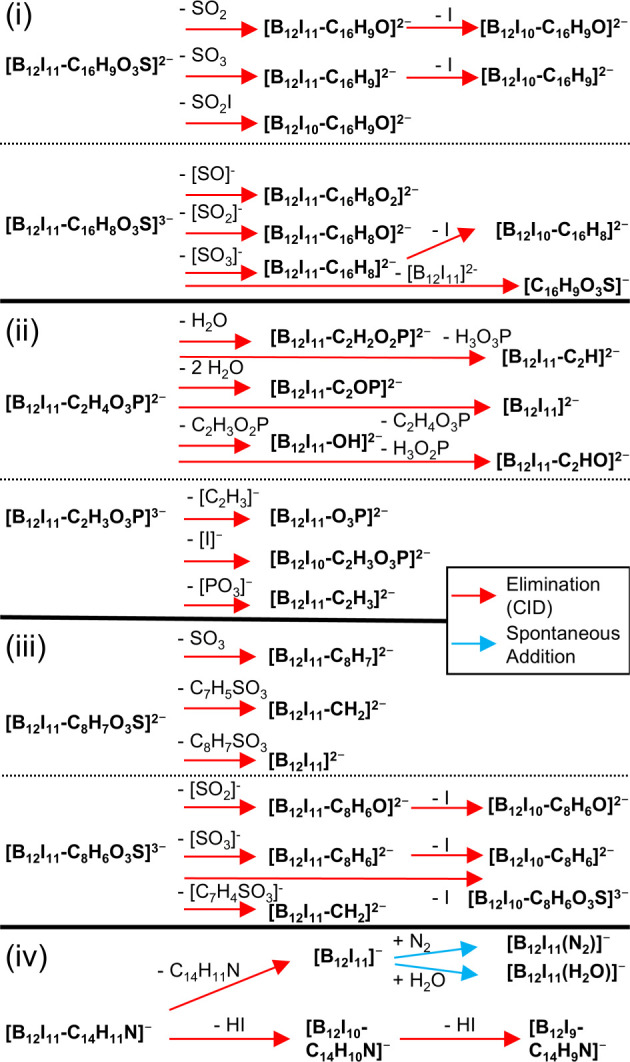
Reactions of the Adducts of [B_12_I_11_]^−^ and Ions (i)–(iv) after Isolation and Fragmentation in an Ion Trap during LESA-MS Analysis of the Dissolved Surface Layer^a^

a For ions (i)–(iii), adducts in charge states −2 and −3 were formed and were investigated. Red arrows indicate elimination reactions; blue arrows indicate spontaneous addition reactions with background molecules of the ion trap. All red arrows correspond to reactions that were individually observed, i.e., arrows originating from the isolated adduct point to MS^2^ products, and those originating from such MS^2^ products point to MS^3^ products. The corresponding MS^2^ spectra of the −3 adducts can be found in Figure 2, and all data on the −2 adducts are shown in the Supporting Information, Figure S17.

The reaction of [B_12_I_11_]^−^ with cation (iv) also results in the substitution of a proton in (iv) by [B_12_I_11_]^−^, but the evaluation of the product ion mass spectrum is challenging. The deuteron of the acidic D–C≡C–R group of ion (iv) was found to exchange with H^+^ in solution over time, and an almost equal ratio of ion (iv) with H–C≡C– and D–C≡C– groups was deposited in our experiments (see +ESI analysis in the Supporting Information, Figure S18). Since the H/D–C≡C group is the most exposed C–H group, we hypothesize that substitution by [B_12_I_11_]^−^ should occur at this position. However, the signal of the product [B_12_I_11_–C≡C–C_12_H_11_N]^−^ overlaps with the signal of ion pairs [H/D–C≡C–C_12_H_11_N]^+^[B_12_I_11_H]^2–^. [B_12_I_11_H]^2–^ is an abundant side product in [B_12_I_11_]^−^ deposition experiments, forming independently of the provided reagent.^39^ Note that the *m*/*z* of such ion pairs is only 2–3 units above that of the expected product [B_12_I_11_–C≡C–C_12_H_11_N]^−^, resulting in strongly overlapping isotopic patterns. Therefore, we fitted the acquired pattern with a combination of simulated patterns: (1) the ion pair [H/D–C≡C–C_12_H_11_N]^+^[B_12_I_11_H]^2–^ with a fixed H/D ratio of 1:1, (2) the expected product [B_12_I_11_–C≡C–C_12_H_11_N]^−^, and (3) [D–C≡C–C_12_H_10_NB_12_I_11_]^−^, which would result from proton substitution at another C–H bond of ion (iv). The best fit was obtained for 92% ion pair (1) and 8% of the expected product (2). No contribution of product (3) was found, confirming the hypothesis that proton substitution indeed occurs in the exposed H/D–C≡C–R group. CID of the ion mixture indicated that the ion pair fragments at a much lower collision energy than the product [B_12_I_11_–C≡C–C_12_H_11_N]^−^. After applying moderate excitation energy (CID energy of 25 (vendor-specific units)), a narrower ion pattern centered at *m*/*z* 1719 was observed, which showed a different fragmentation behavior than the ion pair-dominated mixture at a lower collision energy (see Supporting Information, Figure S19). The best fit of the remaining signal indicated contributions of roughly 70% of product (2) (see more details in the Supporting Information). Therefore, the results suggest that substitution of a proton/deuteron at the H/D–C≡C–R group is the dominant binding motif of [B_12_I_11_]^−^ for ion (iv).

Herein, we did not probe the behavior of [B_12_X_11_]^−^ binding with branched hydrocarbons at the interfaces. However, previous studies on the functionalization of the dipeptide leucyl proline (which contains a 2-methylbutyl alkyl chain) with [B_12_I_11_]^−^ confirmed that proton substitution is also the dominant reaction for this substance class.^9^

### 3.3 Comparison of Reactions in the Gas Phase and at Vacuum/Solid Interfaces

In the gas phase, only the reaction with terminal methyl groups leads to substitution of a proton via B–C bond formation. Only in such cases, an additional water adduct was observed, resulting in the formation of [H_3_O]^+^[B_12_Br_11_–CH_2_–C_*n*_H_2*n*+1_]^2–^ due to the strong Brønsted acidity of the proton Coulombically bound to the borate anion. Selected examples of this reaction are shown in the Supporting Information (Figure S20).^51^ In contrast, double and triple bonds are strongly bound to [B_12_X_11_]^−^ ions via direct addition to the electrophilic vacant boron atom without cleaving a C–H bond. Reactions with aromatic units result in adduct formation. From such adducts, the reactants are regenerated upon excitation. However, at layer interfaces, proton substitution occurs not only at aliphatic groups but also at double and triple bonds and aryl compounds, as indicated by the analysis of the products formed upon ion deposition.

### 3.4 Computational Investigations and Conceptual Model Development

To develop a rational model explaining the results of both gas-phase and fragment ion deposition experiments, DFT investigations were carried out to determine the preferred binding mode in the gas phase. Enthalpies and transition states (TS) were investigated for the rearrangement of the initially formed direct adduct (DA) into proton substitution-binding mode. The results are shown in Figure 3. Note that Figure 3 does not display barriers or enthalpy changes for fragmentation reactions. Computational results indicate that, in most cases, the minimum-enthalpy structure correlates well with the binding mode indicated by CID experiments shown in Figure 1. In the case of aromatic compounds (benzene and toluene, Figure 3a and b), the aryl-bound DA with intact C–C and C–H bonds in the aromatic compound is thermochemically preferred. Electron density is provided to the vacant boron atom from the π-system, similar to the case of protonated aryl compounds.^52^ This binding mode enables fast dissociation into the reactants upon excitation by reforming the aromatic electron configuration in the hydrocarbon. In the case of an alkene (1-butene, Figure 3c), binding to the double bond via B–C bond formation is strongly favored over substitution of an alkyl proton. The strong interactions with the double bond result in the formation of [B_12_Br_11_(C_2_H_4_)]^−^ upon fragmentation. In the case of a branched hydrocarbon with a proton on a tertiary carbon atom (2-methylbutane, Figure 3d), a high-lying TS hinders proton substitution from this binding position. Therefore, hydride abstraction during CID can be rationalized from the B–H–C_*tert*_ structure motif.^6^ In the case of cyclic alkenes, DFT predicts direct binding via the double bond (cyclohexene, Figure 3e), but CID results in hydride abstraction (see Figure 1) similarly to that of compounds bound via a tertiary C–H bond. We hypothesize that the formation of [B_12_Br_11_(C_2_H_4_)]^−^ upon CID—a typical fragment ion of a linear alkene adduct—is unfavorable for a cyclic alkene because two covalent bonds would need to be broken. Instead, the observed fragmentation behavior may be governed by rearrangement into an allyl-bound isomer followed by dissociation. The fragmentation of both B–H–C_*tert*_- and allyl-bound isomers via hydride abstraction followed by proton transfer (as indicated by the detected product ion pair [B_12_Br_11_H]^2–^[H]^+^) may be favored due to the formation of a stabilized carbocation in the first step. Similar to cyclic alkenes, adducts with C=C bonds conjugated to an aryl unit do not yield [B_12_Br_11_(C_2_H_4_)]^−^ upon CID, although the double-bond-bound adduct is favored thermodynamically over the aryl-bound adduct. The potential energy surface (PES) for the styrene adduct is shown in Supporting Information and Figure S21. Here, rearrangement into the aryl-bound adduct is apparently favored over H atom transfer from the aromatic ring, explaining the exclusive observation of dissociation into the reactants upon the CID of the adduct (Figure 1). In the case of a triple bond reagent like phenylacetylene (Figure 3f), the formation of the triple bond-bound adduct is strongly favored over the aryl-bound adduct, and it is also enthalpically more favorable than proton substitution. The deep energetic sink of this adduct rationalizes why HBr elimination becomes the most favorable pathway upon CID. PES diagrams calculated for the reactions of [B_12_I_11_]^−^ with benzene and toluene are shown in the Supporting Information (Figure S22) and demonstrate that the fundamental reactivity is independent of the halogen substituent (X) of the borate anion.

**Figure 3:**
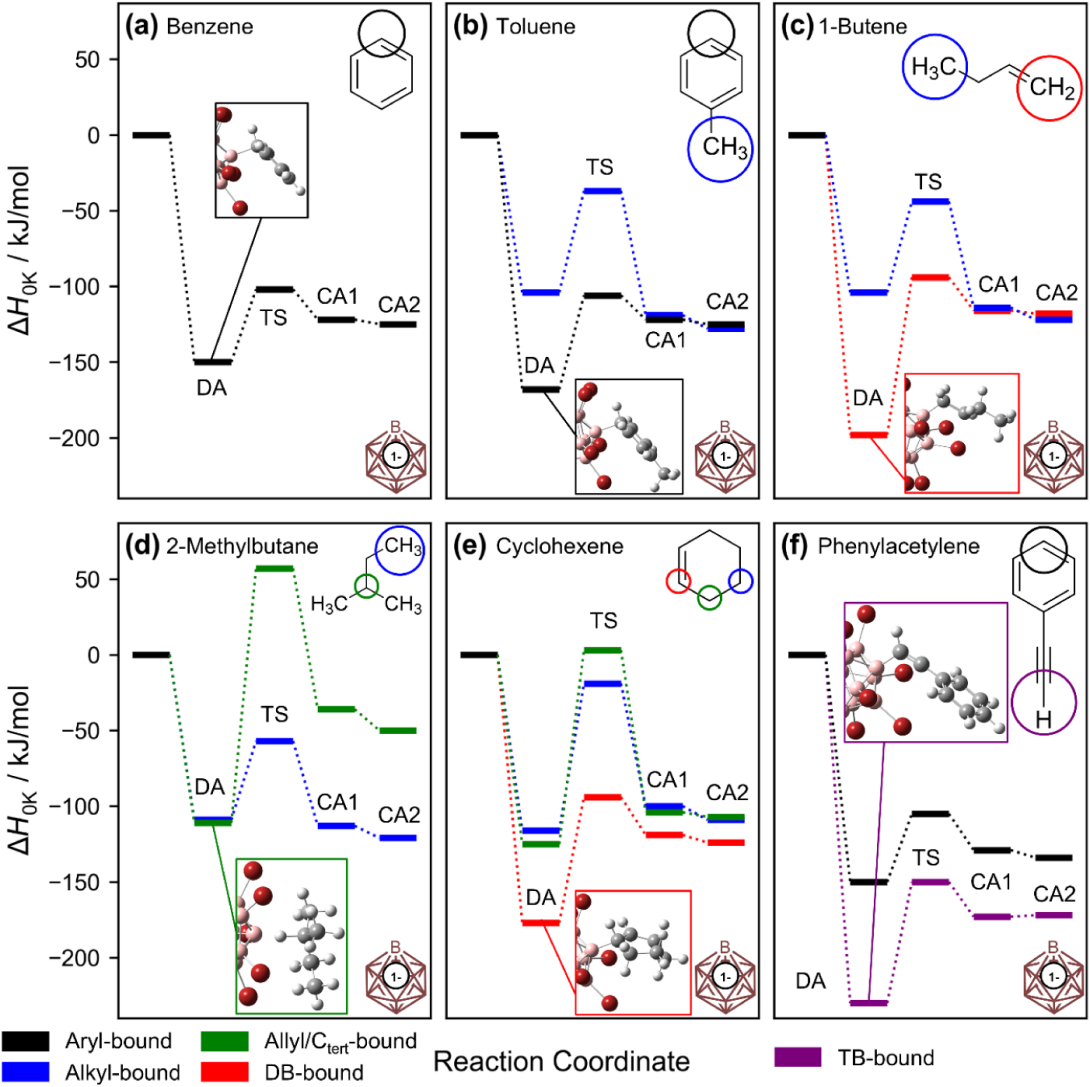
PES diagrams calculated (B3LYP-D3BJ/def2-TZVPP) for adduct formation and subsequent proton substitution by [B_12_Br_11_]^−^ with (a) benzene, (b) toluene, (c) 1-butene, (d) 2-methylbutane, (e) cyclohexene, and (f) phenylacetylene. Each investigated reaction proceeds via the formation of a direct adduct (DA), in which all of the C–H bonds of the hydrocarbon are still intact. The formation of the B–C bond leading to the formation of a covalent adduct (CA1) proceeds via a transition state (TS). Note that initially formed CA1, with a B–C bond, is typically not the enthalpically most stable structure. The substituted proton can move across Br–Br edges of the ion, and typically, a remote edge represents the most favorable geometry, CA2. Intermediate steps are omitted from the PES diagrams shown; a detailed discussion also involving transition states for the proton movement can be found in ref 5. Color codes in Figure 3 are as follows: Black—aryl-bound, blue—alkyl-bound, green—allyl-bound or bound to a tertiary C atom, red—double bond (DB)-bound, and purple—triple bond (TB)-bound. Each panel contains an excerpt from the optimized geometry of the DA minimum.

Computational results rationalize the experimental gas-phase results. However, an explanation for the observed proton substitution at the interface for all probed cases (ions (i)–(iv)) is still required. Although proton substitution is not the most enthalpically favorable reaction path in the gas phase, it is noteworthy that it is thermochemically accessible in all cases; i.e., the energy gained by the formation of the DA between separated [B_12_Br_11_]^−^ and the respective hydrocarbon is sufficient to overcome the TS for proton substitution, irrespective of binding to a terminal alkyl, alkene, aryl, or acetylene group. It is reasonable to assume that different binding modes are populated in the “hot” ion-molecule complex formed upon collision of [B_12_Br_11_]^−^ with the hydrocarbon, including the covalent adduct after proton substitution in Figure 3. Prior to thermalization of the product ion, fast interconversion of the binding modes may be possible. In the interfacial reactions on surfaces, abstracted protons can dissociate from the borate anion and dissipate into the condensed ionic layer. Therefore, this reaction path is strongly favored entropically because a proton is removed from the intramolecular conversion equilibrium between different binding modes (principle of Le Chatelier). The generation of mobile positive-charge carriers within the layer may be additionally favorable for local charge balancing during anion deposition at the interface. In contrast, in the gas phase, the proton cannot escape from the anion due to strong electrostatic attraction. Reverse reactions can occur, and slow thermalization due to collisional cooling results in the enthalpically most favorable binding mode.

We note that a quantitative evaluation of these effects is beyond the scope of this study and would require the consideration of different cooling rates of products in the gas phase and at interfaces as well as the quantitative entropic and enthalpic influences of the condensed phase environment for the PES of the interface reaction.

## 4 Conclusions

The interaction between [B_12_X_11_]^−^ ions and hydrocarbons was investigated both in the gas phase and at vacuum/solid interfaces. The results provide mechanistic insights into the direct binding of the highly reactive [B_12_X_11_]^−^ ion to different hydrocarbons. In the gas phase, the highly electrophilic vacant boron atom forms B–C bonds with *n*-alkyl units via proton substitution but binds directly to double and triple bonds. The adducts exhibit characteristic structure-dependent fragmentation reactions when subjected to CID. Binding to aryl units results in an adduct that dissociates into the reactants upon CID. In contrast, proton substitution was observed in all cases when [B_12_X_11_]^−^ ions reacted with hydrocarbons at interfaces. This is qualitatively explained by the condensed-phase environment allowing the proton to dissipate into the layer, favoring proton substitution entropically.

The obtained insights highlight the possible differences in the binding modes of reactive ions to reagents in the gas phase and at interfaces and form an important basis for the development of analytical and preparative applications of [B_12_X_11_]^−^ ions for charge tagging of nonpolar organic groups. In the gas phase, adducts corresponding to the most thermodynamically favorable binding mode are formed, and fragmentation of these adducts yields structure-dependent characteristic product ions that indicate the binding site. In contrast, reactions at interfaces result in almost structure-independent B–C bond formation via proton substitution at an interface-active C–H bond, forming a C–B_12_X_11_ group, regardless of the degree of hydrocarbon saturation.

## Supplementary Material



## Data Availability

Data for this article including input and output files of computational investigations and raw data files of mass spectrometry investigations are available at ioChem-BD at https://dx.doi.org/10.19061/iochem-bd-6-606 and at PURR Publications at https://purr.purdue.edu/publications/4990/1.

## Notes

Additional experimental details (precursor synthesis, fragment ion deposition), additional complementary gas phase and fragment ion deposition mass spectra for X = Br, I, additional complementary computational data for X = I (PDF)
