# Supplementary material for: Functionalization of Saturated and Unsaturated Hydrocarbons with Electrophilic Anions [B12X11]− (X = Br, I) in the Gas Phase and at Interfaces
Source: J Phys Chem A. 2026 Jan 30;130(6):1364–74. doi: 10.1021/acs.jpca.5c08384 (PMC12908116; doi:10.1021/acs.jpca.5c08384)
Supplement: Supplementary file 1 [file jp5c08384_si_001.pdf]

Supporting Information

to

Functionalization of Saturated and Unsaturated Hydrocarbons with Electrophilic Anions $[B_{12}X_{11}]^-$ (X=Br, I) in the Gas Phase and at Interfaces

Markus Rohdenburg,[1] Jaskiran Kaur,[2] Ashley J. Galligan,[2] Aby-Paul Benny,[1] Kay-Antonio Behrend,[1] Xin Ma,[3] Stanislav Petrovskii,[4] Kirill Monakhov,[4] Hilkka I. Kenttämaa,[2] Jonas Warneke[1,4*]

*Correspondence should be addressed to: jonas.warneke@uni-leipzig.de

[1] Wilhelm-Ostwald-Institut für Physikalische und Theoretische Chemie, Universität Leipzig, Linnéstr. 2, 04103 Leipzig, Germany.

[2] James Tarpo Jr. and Margaret Tarpo Department of Chemistry, Purdue University, West Lafayette, 560 Oval Drive, IN, 47907, USA.

[3] Department of Chemistry, University of Virginia, 409 McCormick Rd, Charlottesville, VA, 22904.

[4] Leibniz Institute of Surface Engineering (IOM), Permoserstr. 15, 04318, Leipzig, Germany.

Contents

Synthesis of the precursor salt for ion (iv) S3
Additional experimental details on fragment ion deposition S9
Kinetic energy measurements S11
Fragment spectra for gas-phase adducts of $[B_{12}I_{11}]^-$ S17
Additional fragment spectra for gas-phase adducts of $[B_{12}Br_{11}]^-$ S18
Fragment ion deposition with $[B_{12}Br_{11}]^-$ S19
Additional MS^2 spectra for (2-) adducts of ions (i)-(iii) and (1-) adduct of ion (iv) S20
Determination of the D/H ratio in ion (iv) S21
Fit of the MS^2 spectrum of the adduct of ion (iv) S22
Brønsted acidity of selected gas-phase adducts S23
PES diagram for $[B_{12}Br_{11}]^-$ reacting with styrene S24
PES diagrams for $[B_{12}I_{11}]^-$ reacting with selected hydrocarbons S25
References S26

Synthesis of the precursor salt for ion (iv)

The solvents (THF, toluene, CH_2Cl_2) for the synthesis of deuterated compounds were purified and dried according to the standard protocols.[1]

The synthesis of ion (iv) in the form of its trifluoromethanesulfonate salt was performed according to Scheme S1.

4-(4-ethynylphenyl)pyridine was synthetized according to the previously published procedure.[2]

4-(4-(ethynyl-D)phenyl)pyridine was synthetized employing the adopted approach described for phenylacetylene deuteration.[3] A Schlenk flask was charged with 301 mg (1.68 mmol) of 4-(4-ethynylphenyl)pyridine, 8 mL of dry toluene and 5 mL of dry THF. The solution was degassed, cooled to – 30°C, and 1.2 mL of 1.6 M solution of *n*-BuLi (1.92 mmol) in hexanes was added dropwise with vigorous stirring. The addition of *n*-BuLi resulted in gelatinous mass, which was turned to a clear solution by additional 5 mL of THF. To this solution 2 mL of D_2O was added, and the system was stirred vigorously 10 minutes at room temperature. The organic layer was separated, evaporated to dryness and the crude product was separated with a column chromatography on silica gel. The fractions with R_f(CH_2Cl_2/EtOAc/EtOH 4/1/0.05) = 0.4 were combined and dried *in vacuo*. Yield: 146 mg (48.2%); off-white solid. ^{1}H NMR (600 MHz, Chloroform-*d*) δ 8.67 (d, *J* = 5.0 Hz, 2H), 7.63 – 7.57 (s, 4H), 7.52 – 7.47 (m, 2H), 3.18 (s, 0.25H). ^{1}H NMR (600 MHz, Chloroform-*d*) *δ* 8.67 (d, *J* = 5.0 Hz, 2H), 7.63 – 7.57 (m, 4H), 7.52 – 7.47 (m, 2H), 3.18 (s, 0.25H). ^{13}C NMR (151 MHz, Chloroform-*d*) *δ* 150.46, 147.63, 138.52, 133.04, 127.11, 123.19, 121.69, 82.76, 78.98. The assignment of ^{1}H and ^{13}C NMR peaks is presented in Fig. S1 and S2. An additional HSQC spectrum is shown in Fig. S3. The NMR data indicate that the product contains approx. 25% H and 75% D adjacent to acetylene moiety.

4-(4-(ethynyl-D)phenyl)-1-methylpyridinium trifluoromethanesulfonate. 123 mg (0.68 mmol) of 4-(4-(ethynyl-D)phenyl)pyridine) was dissolved in 3 mL of dry CH_2Cl_2, flushed with argon and cooled to - 10°C. 250 µL (2.27 mmol) of methyl trifluoromethanesulfonate was added dropwise with the intense stirring. The cooling bath was removed, and the mixture was stirred additionally 30 minutes. The white precipitate was collected, washed with 7 mL of CH_2Cl_2 and 7 mL Et_2O, and dried *in vacuo*. Yield: 110 mg (49 %); off-white solid. ^{1}H NMR (400 MHz, Methanol-d_4) *δ* 8.90 (d, *J* = 6.8 Hz, 2H), 8.40 (d, *J* = 6.9 Hz, 2H), 8.01 (d, *J* = 8.5 Hz, 2H), 7.72 (d, *J* = 8.4 Hz, 2H), 4.42 (s, 3H), 3.83 (s, 0.32H). ^{13}C NMR (101 MHz, Methanol-d_4) *δ* 155.19, 145.40, 133.89, 132.90, 127.83, 126.45, 124.47, 120.15 (q, J_{C-F} = 318.2 Hz), 81.91, 81.02, 46.70. The assignment of ^{1}H and ^{13}C NMR peaks is presented on Fig. S4 and S5. Additional HSQC and HMBC spectra are shown in Fig. S6 and S7, respectively. The NMR data indicate that the product contains approx. 32% H and 68% D adjacent to acetylene moiety.

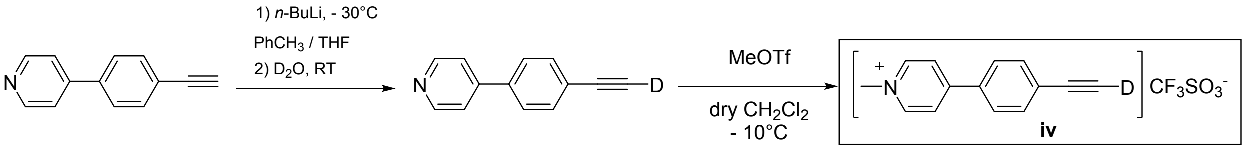

Scheme S1. Synthesis of the precursor salt for ion (iv).

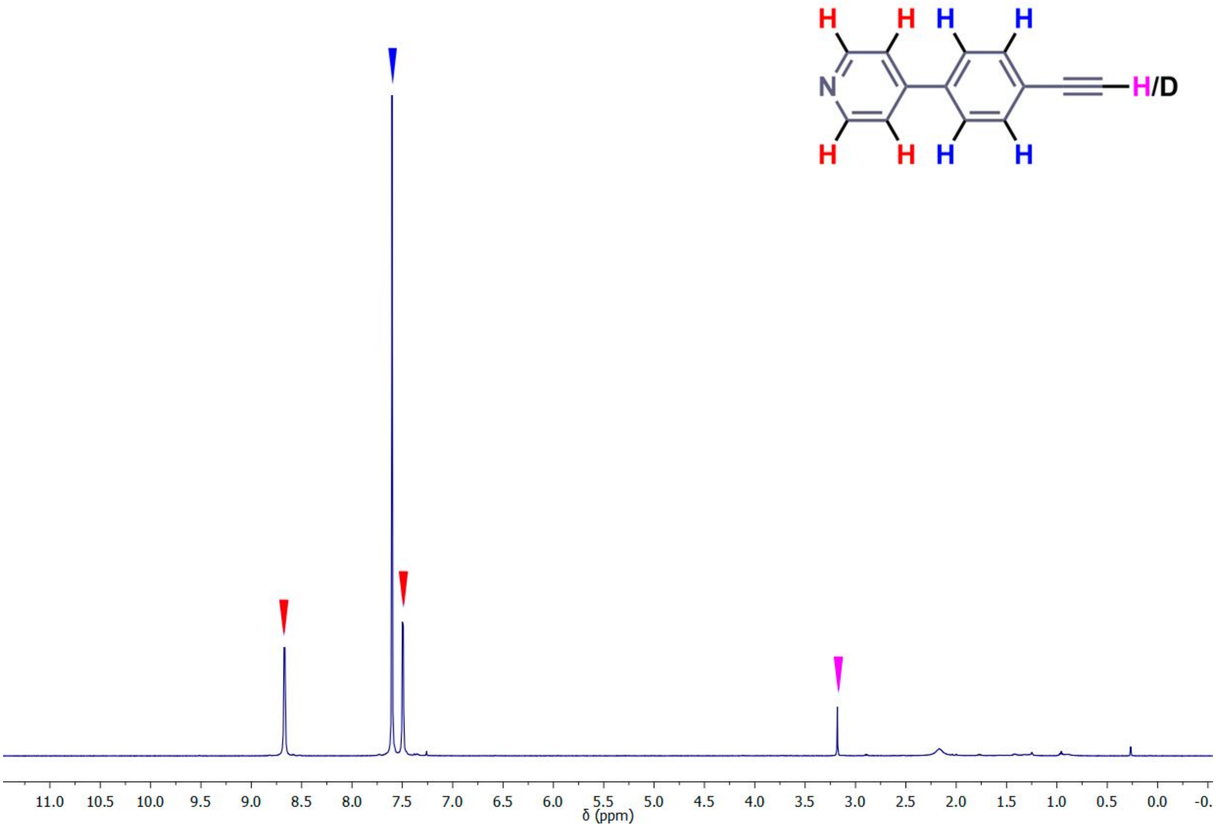

Figure S1. ^{1}H NMR spectrum (600 MHz, Chloroform-*d*) of 4-(4-(ethynyl-D)phenyl)pyridine.

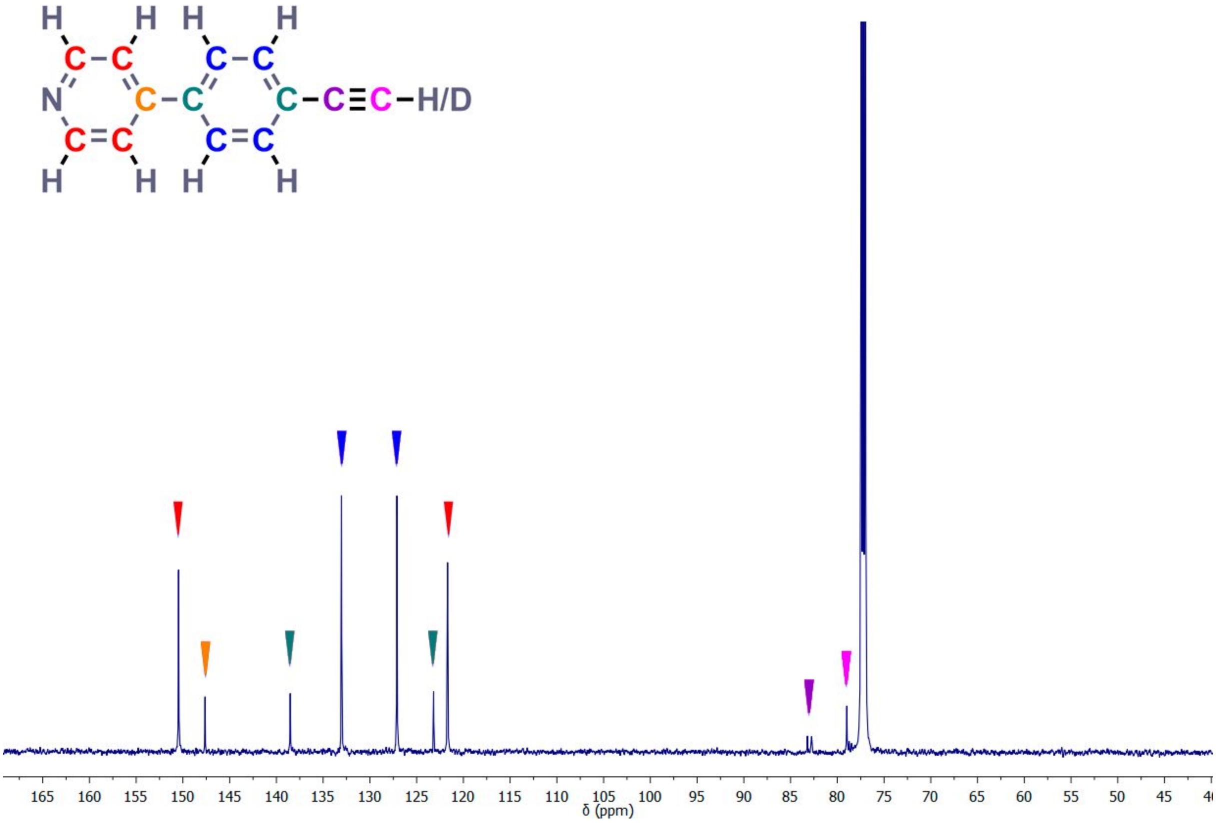

Figure S2. ^{13}C NMR spectrum (151 MHz, Chloroform-*d*) of 4-(4-(ethynyl-D)phenyl)pyridine.

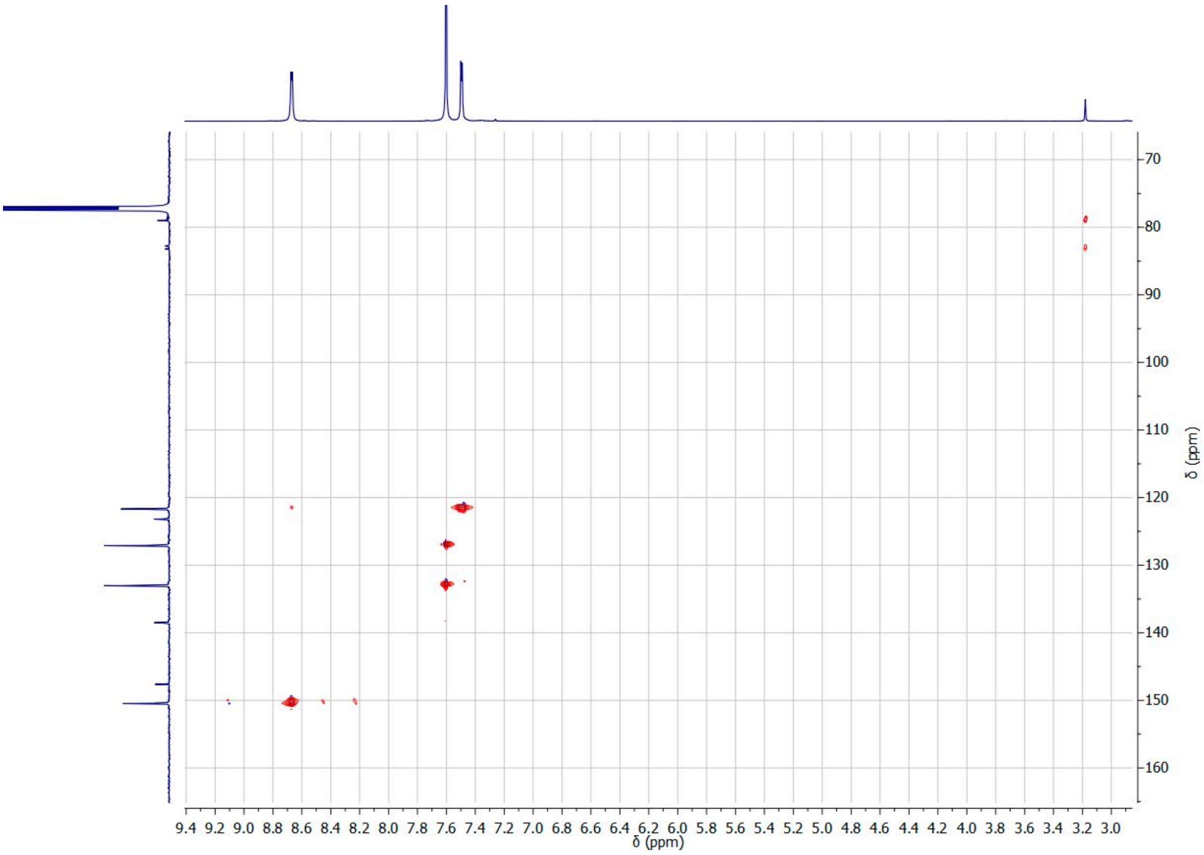

Figure S3: HSQC spectrum of 4-(4-(ethynyl-D)phenyl)pyridine.

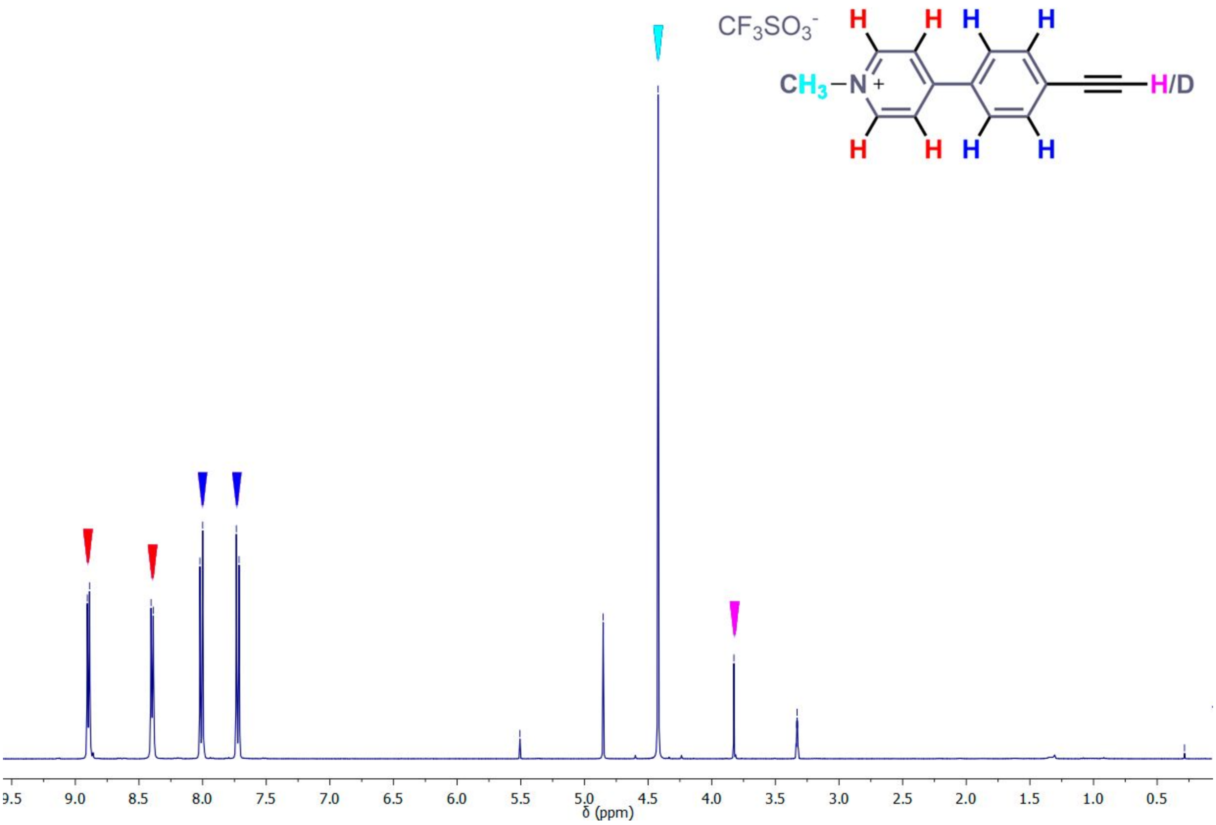

Figure S4. ^{1}H NMR spectrum (400 MHz, Methanol-d_4) of 4-(4-(ethynyl-D)phenyl)-1-methylpyridinium trifluoromethanesulfonate.

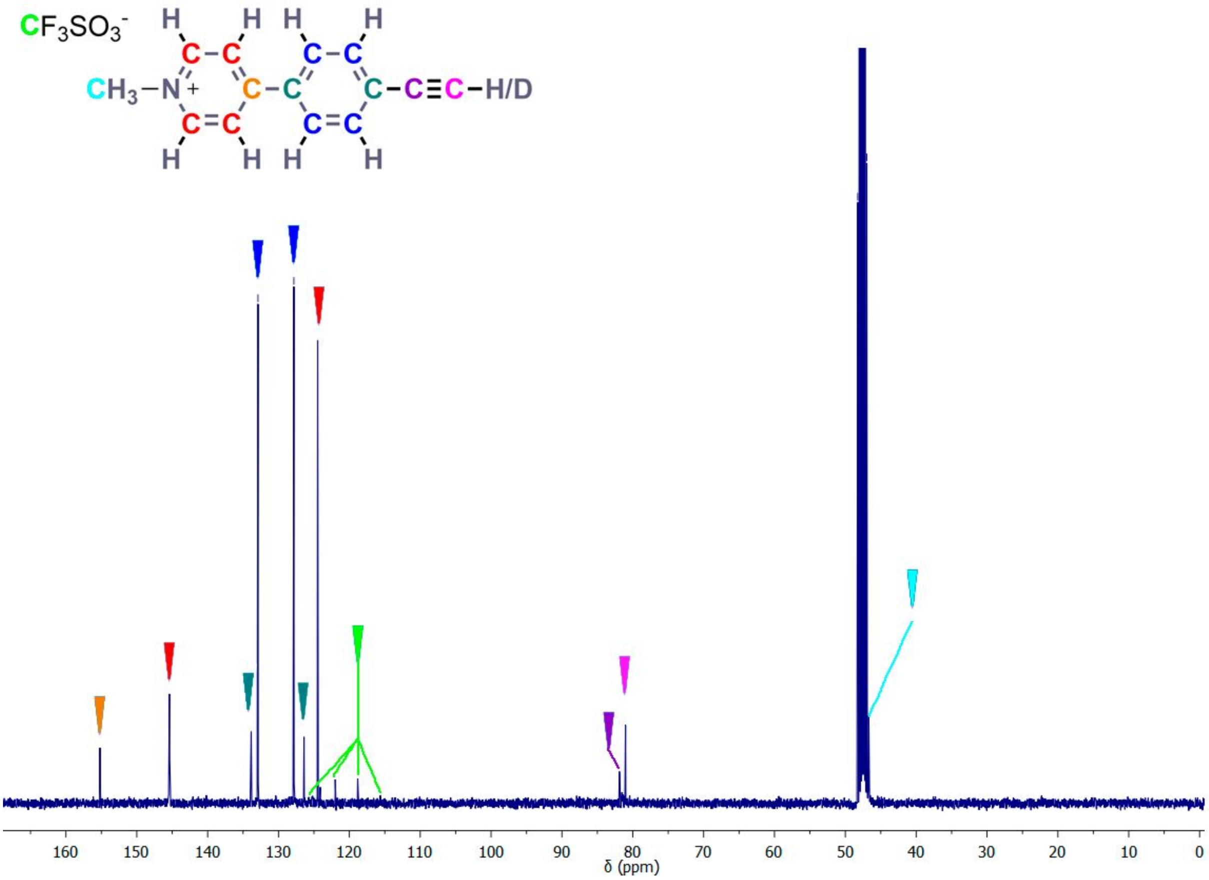

Figure S5. ^{13}C NMR spectrum (101 MHz, Methanol-d_4) of 4-(4-(ethynyl-D)phenyl)-1-methylpyridinium trifluoromethanesulfonate.

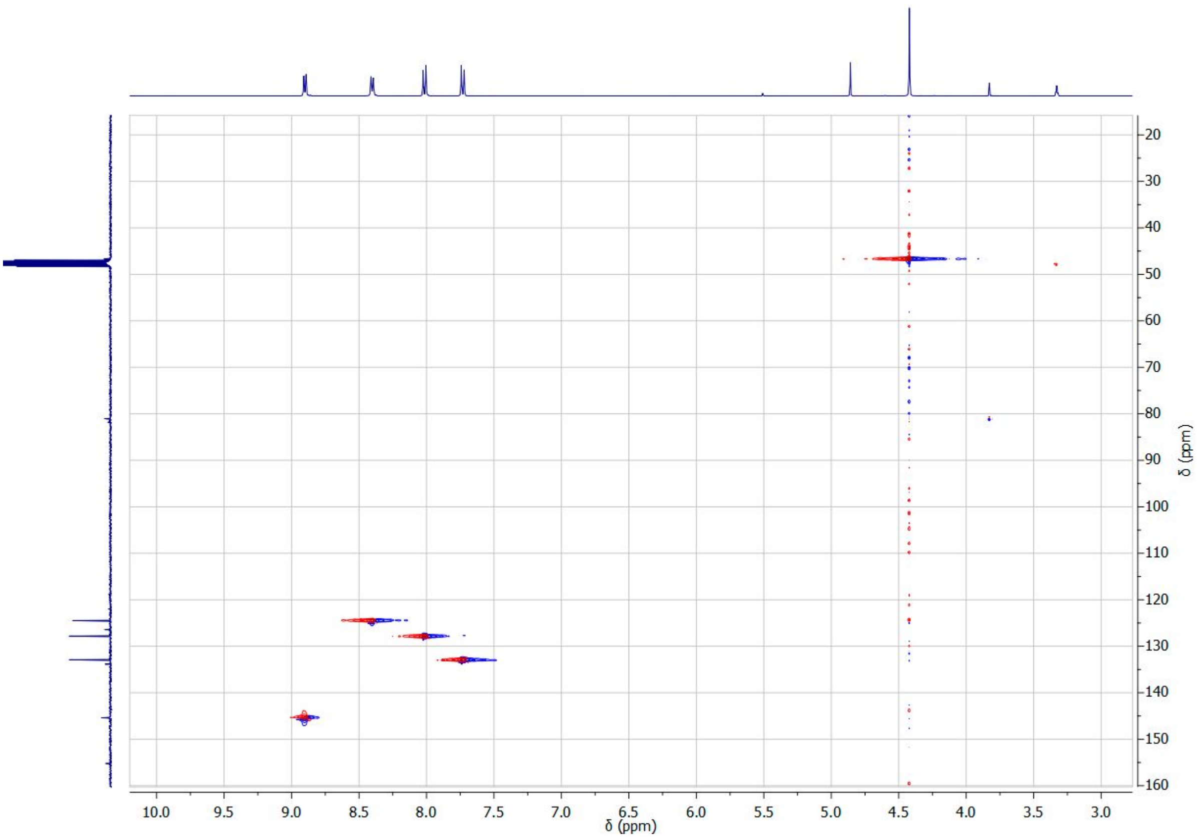

Figure S6. HSQC spectrum of 4-(4-(ethynyl-D)phenyl)-1-methylpyridinium trifluoromethanesulfonate.

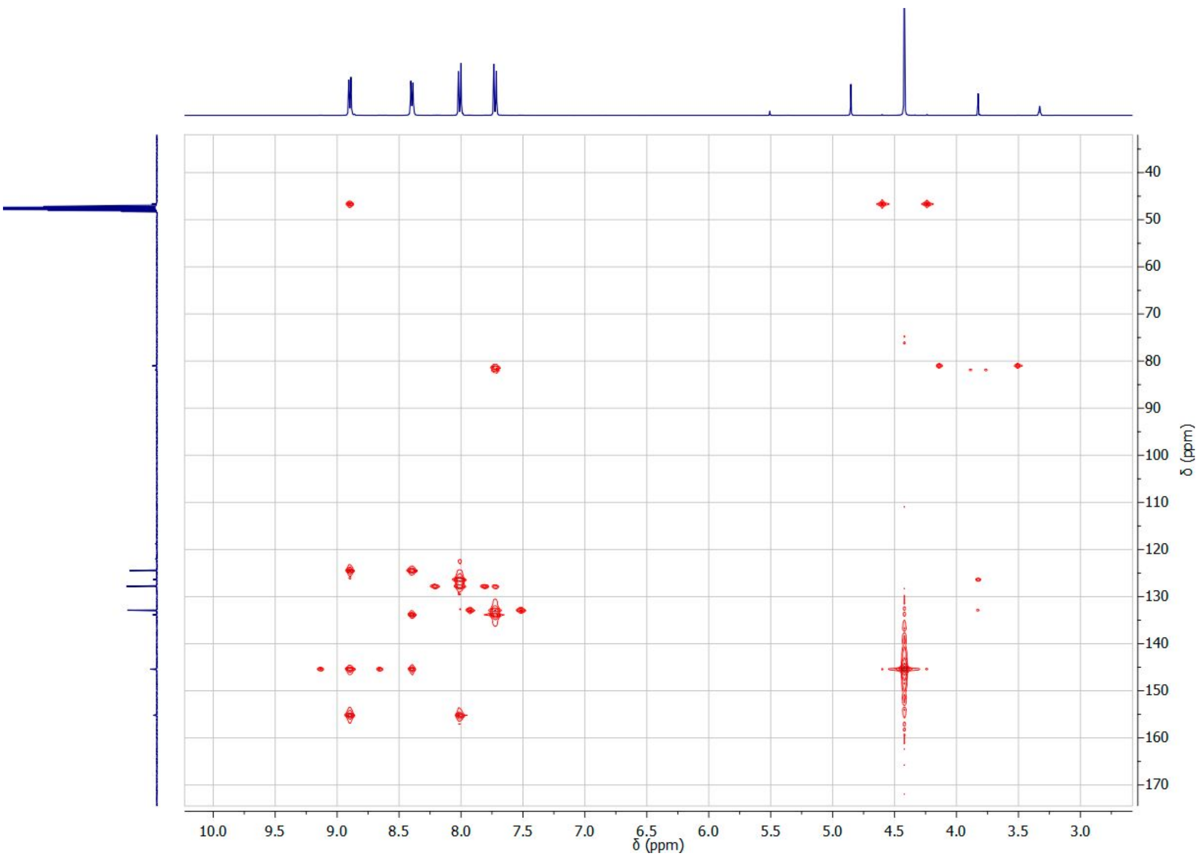

Figure S7. HMBC spectrum of 4-(4-(ethynyl-D)phenyl)-1-methylpyridinium trifluoromethanesulfonate.

Additional experimental details on fragment ion deposition

The two electrospray ionization (ESI) sources of a previously described ion soft-landing instrument optimized for fragment ion deposition[4,5] were used to transfer the ions from solution into the gas phase. 0.1 mM solutions of $K_2[B_{12}Br_{12}]$ or $K_2[B_{12}I_{12}]$ in acetonitrile (ACN) were used as the precursor solutions for the generation of the reactive fragment $[B_{12}X_{11}]^-$. In addition, 0.1-0.2 mM solutions of salts containing the corresponding organic ions (i)-(iv) in ACN were used for cation co-deposition experiments. Two ion funnels operating at high and low pressure (high-pressure ion funnel (HPF) and low-pressure ion funnel (LPF)), respectively, focused the ion beam and transferred the gaseous ions from the source region to a rough vacuum stage. Downstream of the ion funnels, CID of the precursor ions was carried out in a collision cell (CC) by applying a voltage difference to ion optics elements. Multiple collisions with background gas molecules lead to fragmentation reactions. The use of a 90° bent ion guide (BIG) enabled the separation of the ion beam from the stream of neutral molecules originating from the source. Ions were then selected by a quadrupole mass filter (QMF) based on their mass-to-charge ratio and guided to the deposition surface, either directly or through a series of einzel lenses and an octupole ion guide, as described previously.[4] In front of the deposition surfaces, a series of three lenses was mounted to focus the ion beam, to which typically voltages of (-)300 V, (-)100 V and (-)300 V were applied, as described previously.[6] The deposition surface consisted of a silicon wafer (1 × 1 cm^2) covered with a 30 nm thick gold adlayer that is bound to the underlying Si surface *via* a 5 nm thick chromium adhesion layer (Siegert Wafer GmbH, Aachen, Germany). Prior to soft-landing experiments, the surface was cleaned by successively immersing the sample in Millipore water and high purity ethanol under ultrasonication for at least five minutes. Subsequently, remaining contaminations were removed by combined UV/ozone cleaning (Ossila UV Ozone Cleaner L2002A, Ossila Limited, Sheffield, UK). The conductive gold surface was grounded *via* a picoammeter, which measured the current of soft-landed ions during the deposition. The number of deposited ions was derived by integrating the ion current over time. The ion optics are powered and controlled by the MIPS control system developed by GAA Custom Electronics, LLC and were adjusted to obtain a maximum ion current after mass selection. Applied RF voltages were adjusted to the resonance frequency of the electric LC circuit. Table S1 gives example settings used during ion deposition.

Table S1. Settings/conditions applied during fragment ion deposition.

Parameter	$[B_{12}Br_{11}]^{-}$	$[B_{12}I_{11}]^{-}$	Anion (i)	Anion (ii)	Anion (iii)	Cation (iv)
**Precursor**	$K_2[B_{12}Br_{12}]$	$K_2[B_{12}I_{12}]$	$C_{16}H_9NaO_3S$	$C_2H_3PO_3H_2$	$C_8H_7NaO_3S$	$[C_{14}H_{11}DN][CF_3SO_3]$
**c (solution) / mM**	0.1	0.1	0.2	0.2	0.2	0.2
**flow rate / mL/h**	0.12	0.12	0.12	0.12	0.12	0.12
**U (ESI) / V**	-2400	-3000	-3000	-3000	-2500	3400
**T (Inlet 1, 2) / °C**	120	25	120	25	120	120
**U (Inlet 1) / V**	-400	-400	-400	-400	-300	400
**U (Inlet 2) / V**	-400	-400	-400	-400	-300	400
**f (HPF) / kHz**	690	676	690	676	690	690
**U (HPF 1) / V**	-390	-450	-400	-450	-370	390
**U (HPF 2) / V**	-350	-280	-320	-280	-290	390
**U (HPF 3) / V**	-358	-310	-238	-320	-280	390
**U (HPF 4) / V**	-128	-170	-117	-140	-80	115
**p (HPF) / Torr**	7.9	7.3	8.0	7.4	8.0	8.0
**f (LPF) / kHz**	922	881	922	881	922	922
**U (LPF 1) / V**	-131	-170	-67	-120	-115	130
**U (LPF lens) / V**	-110	-120	5	-12	-10	3
**U (LPF 2) / V**	-85	-102	-2	-8	-16	1
**p (LPF) / Torr**	1.4	2.3	1.4	2.3	2.4	2.4
**f (CC) / kHz**	1714	1778	1714	1778	1714	1714
**U (CC bias) / V**	-16	-15	-7.7	-7.0	-19.0	2.0
**U (CC lens) / V**	-4.0	-9.0	-12	-8.5	-23.5	6.0
**f (BIG) / kHz**	1660	1773	1660	1773	1660	1660
**U (BIG bias) / V**	-5.7	8.0	-11.7	-7.4	-21.6	6.0
**U (BIG lens) / V**	18.3	30.0	-8.7	5.0	28.4	2.0
**f (QMF) / kHz**	604	550	604	550	604	604
**U (QMF in) / V**	60	200	140	90	-7	-28
**U (QMF pre) / V**	66	-2	11	-1	-18	-90
**U (QMF post) / V**	28	-1	15	-5	-12	-45
**U (QMF out) / V**	0	3	2.4	23	-5	-50
**U (QMF bias) / V**	92	190	121	107	20	-110
m/z	997.0	1503.0	278.0	101.0	183.0	193.0
**Δ *m/z***	15.0	51.0	22.0	88.0	30.0	30.0
**U (EL1 a) / V**	101	-	60	-	60	-28
**U (EL1 b) / V**	0	-	30	-	-12	-90
**f (OIG) / kHz**	1740	-	1740	-	1740	1740
**U (OIG in) / V**	80	-	37	-	24	-45
**U (OIG bias) / V**	29	-	25	-	14	-50
**U (OIG out) / V**	92	-	73	-	13	-22
**U (EL2 a) / V**	53	-	24	-	58	-26
**U (EL2 b) / V**	13	-	31	-	-10	-5
**p (SL chamber) / mbar**	$2\text{-}4\times10^{-6}$	$1\text{-}3\times10^{-5}$	$2\text{-}4\times10^{-6}$	$1\text{-}3\times10^{-5}$	$2\text{-}4\times10^{-6}$	$2\text{-}4\times10^{-6}$
**KE / eV**	9.3	7.5	12.2	6.9	21.6	5.8

Kinetic energy measurements

Kinetic energy (KE) measurements were performed with the retarding potential method, see previous detailed descriptions.[5,7,8] Below, we show the results for the ions as reported in Table S1. We state the most probable KE as determined from the maximum of the probability density curve that results from integrating the sigmoidal fit of the average of five individual measurements.

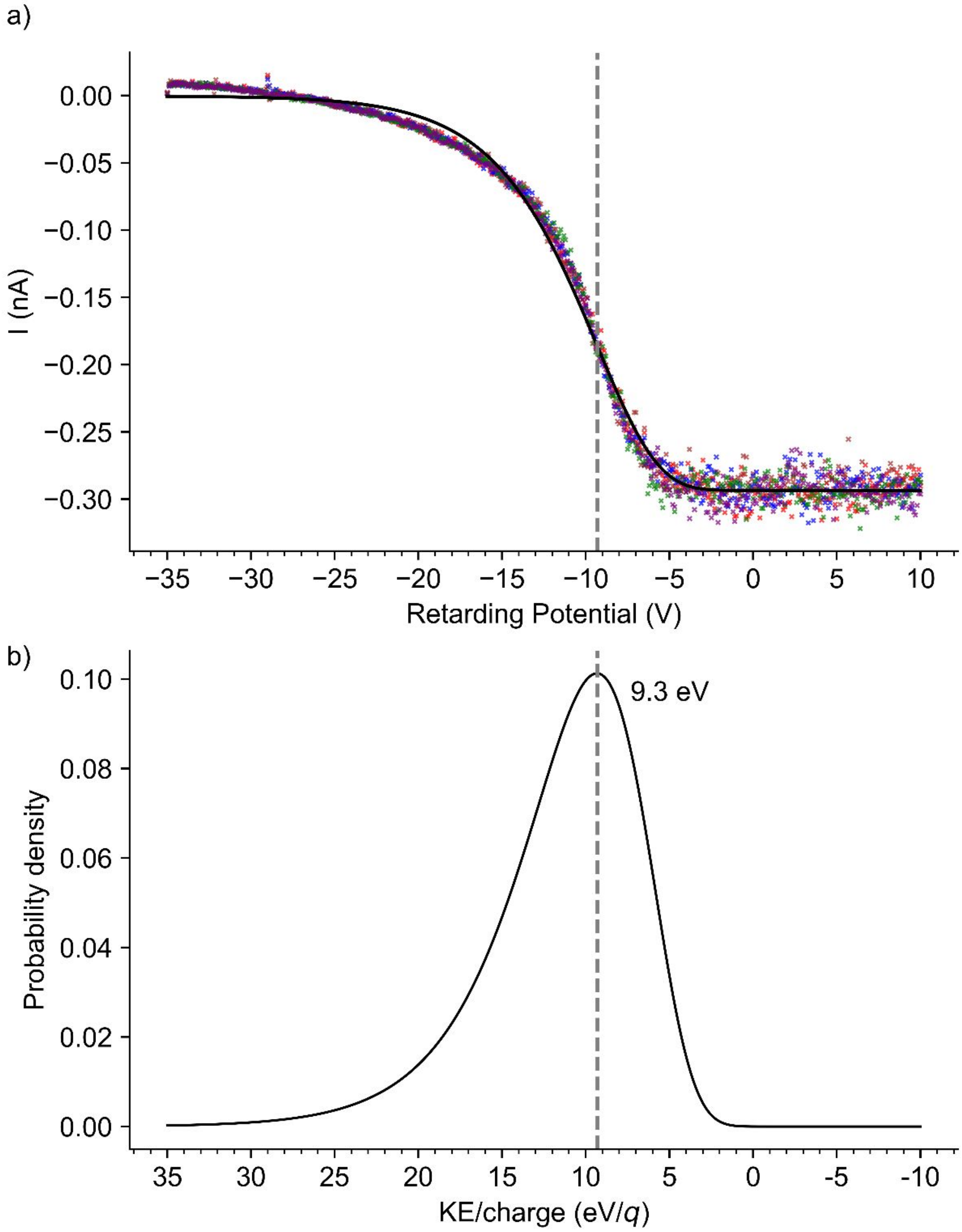

Figure S8. a) Ion current of $[B_{12}Br_{11}]^-$ as a function of the retarding potential. Five separate experiments under identical conditions were performed (individual data points are shown as "X" in a color representative for a single experiment) and all data was subsequently averaged and fitted by a sigmoidal function (black line). The position of the inflection point is marked with a dashed grey line. b) Probability density function for the KE per charge (q) obtained by

calculating the first derivative of the sigmoidal function in a). The position of the maximum is marked with a dashed grey line and can be understood as most probable KE.

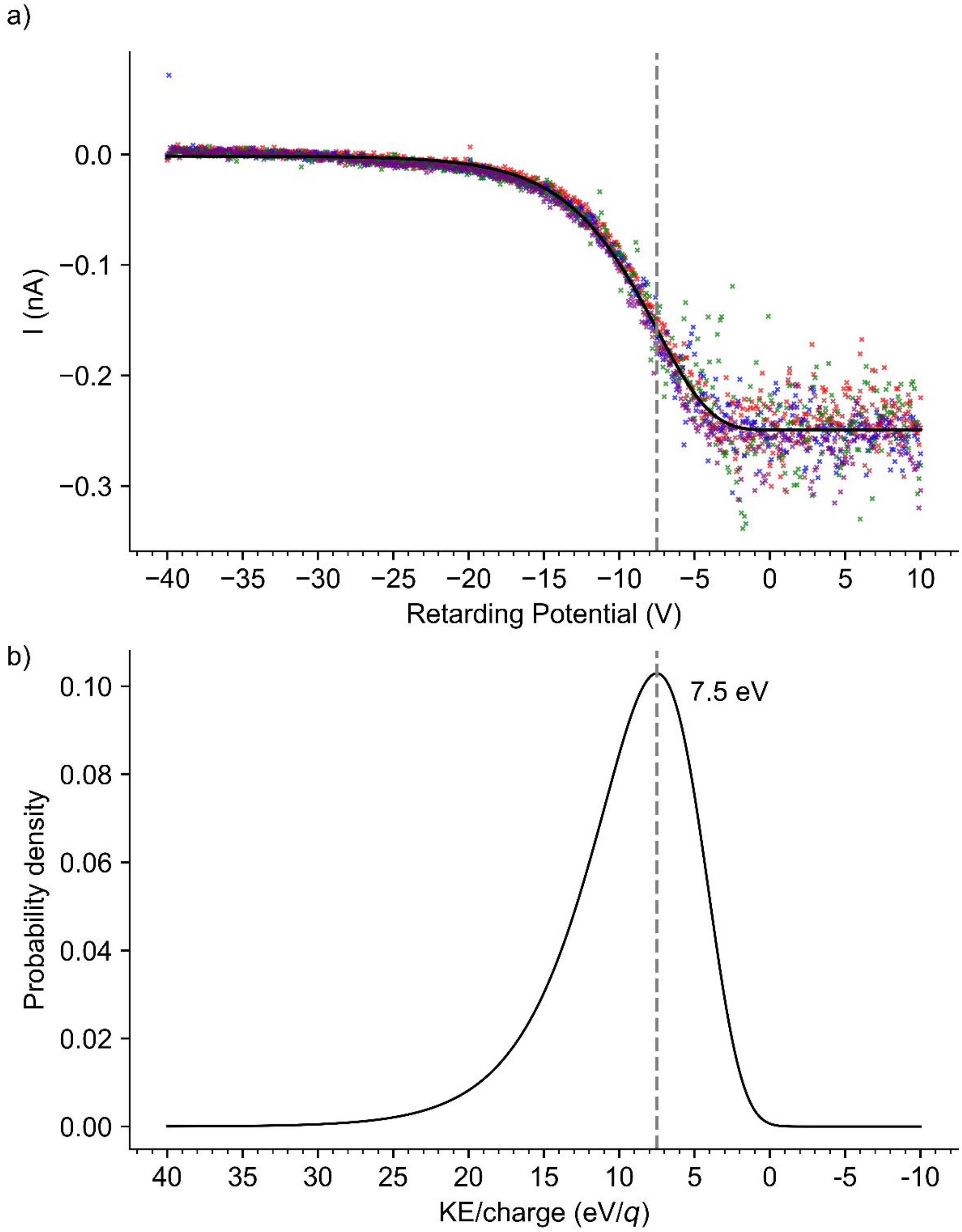

Figure S9. a) Ion current of $[B_{12}I_{11}]^{-}$ as a function of the retarding potential. Five separate experiments under identical conditions were performed (individual data points are shown as "X" in a color representative for a single experiment) and all data was subsequently averaged and fitted by a sigmoidal function (black line). The position of the inflection point is marked with a dashed grey line. b) Probability density function for the KE per charge (q) obtained by calculating the first derivative of the sigmoidal function in a). The position of the maximum is marked with a dashed grey line and can be understood as most probable KE.

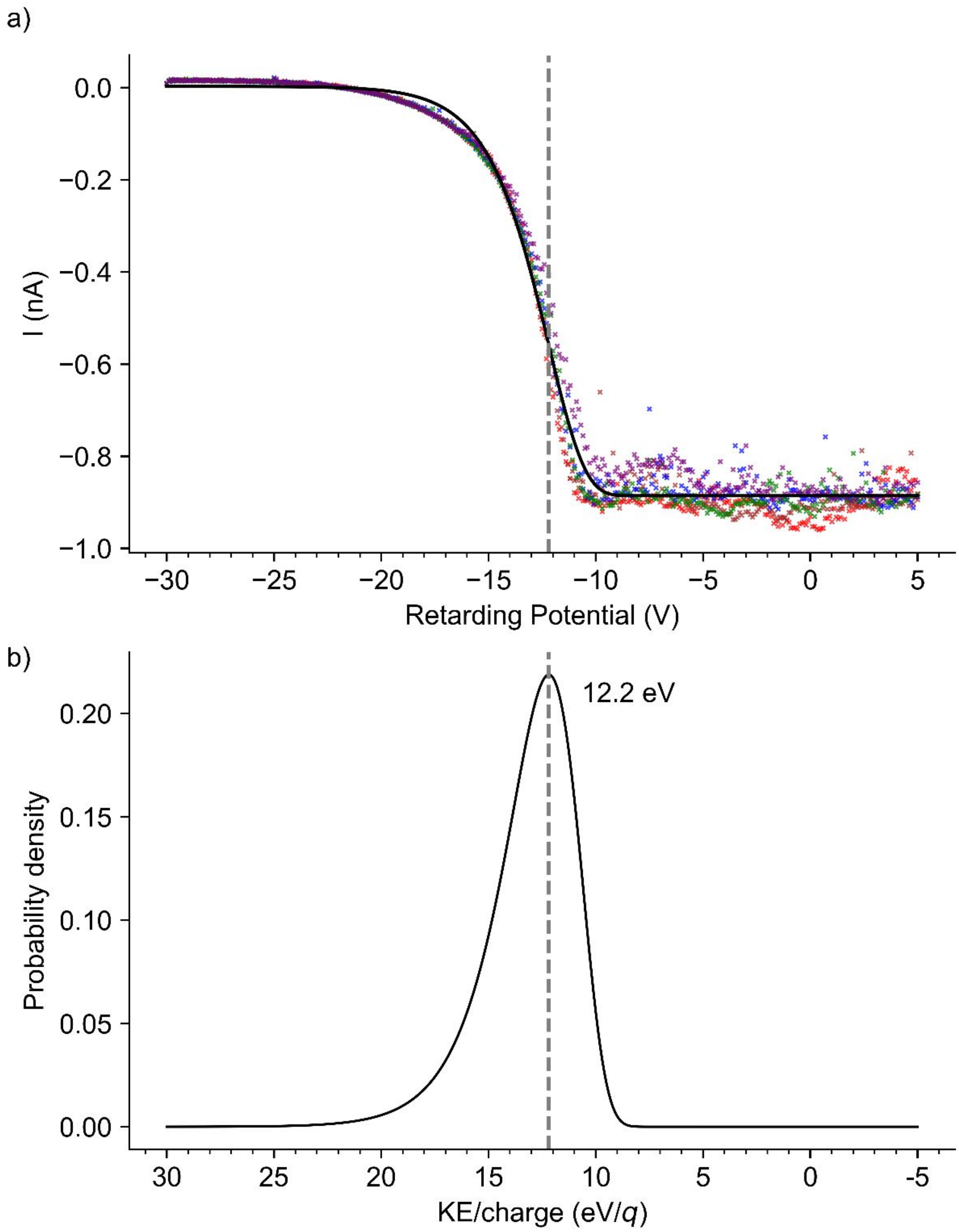

Figure S10. a) Ion current of anion (i) as a function of the retarding potential. Five separate experiments under identical conditions were performed (individual data points are shown as "X" in a color representative for a single experiment) and all data was subsequently averaged and fitted by a sigmoidal function (black line). The position of the inflection point is marked with a dashed grey line. b) Probability density function for the KE per charge (q) obtained by calculating the first derivative of the sigmoidal function in a). The position of the maximum is marked with a dashed grey line and can be understood as most probable KE.

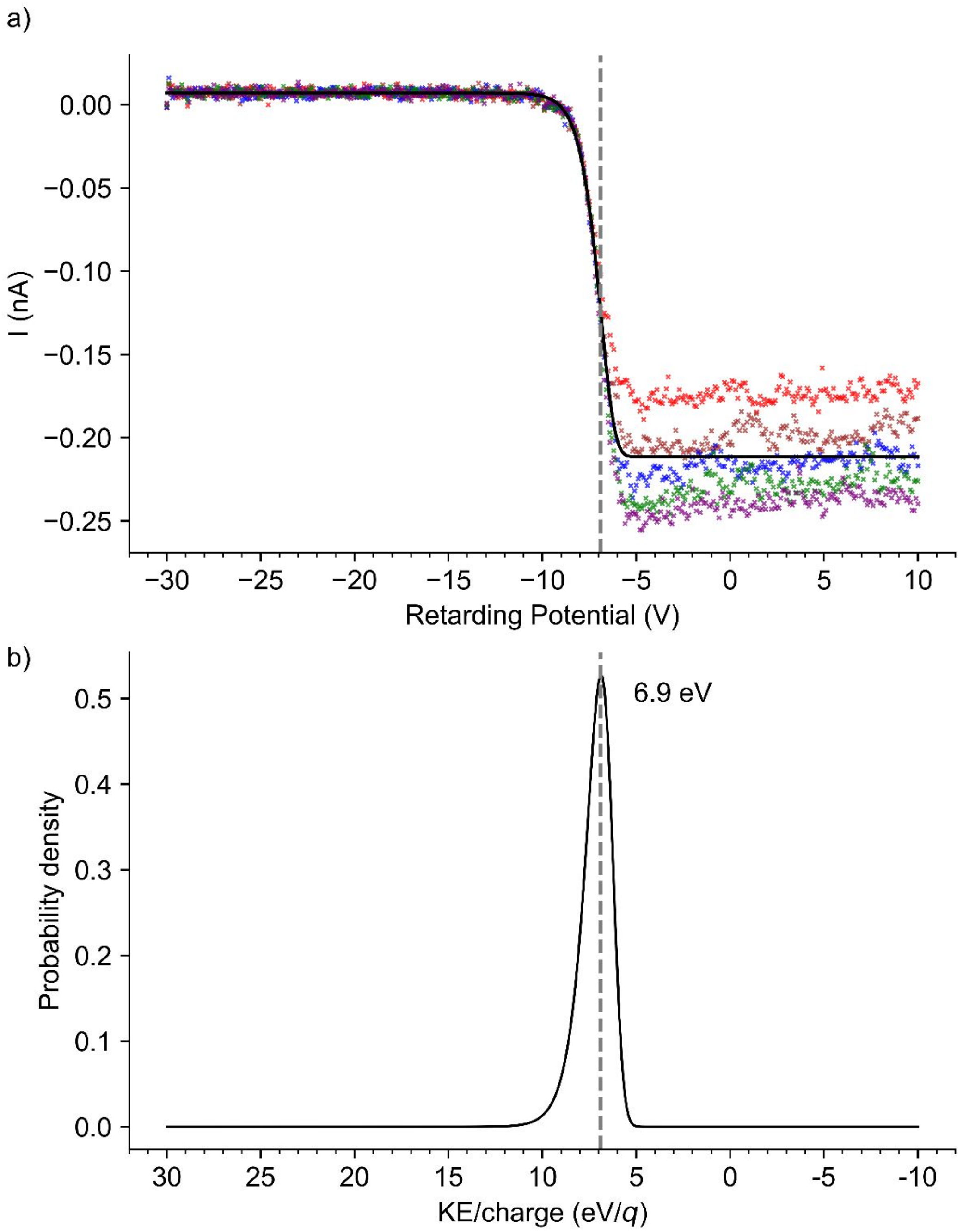

Figure S11. a) Ion current of anion (ii) as a function of the retarding potential. Five separate experiments under identical conditions were performed (individual data points are shown as "X" in a color representative for a single experiment) and all data was subsequently averaged and fitted by a sigmoidal function (black line). The position of the inflection point is marked with a dashed grey line. b) Probability density function for the KE per charge (q) obtained by calculating the first derivative of the sigmoidal function in a). The position of the maximum is marked with a dashed grey line and can be understood as most probable KE.

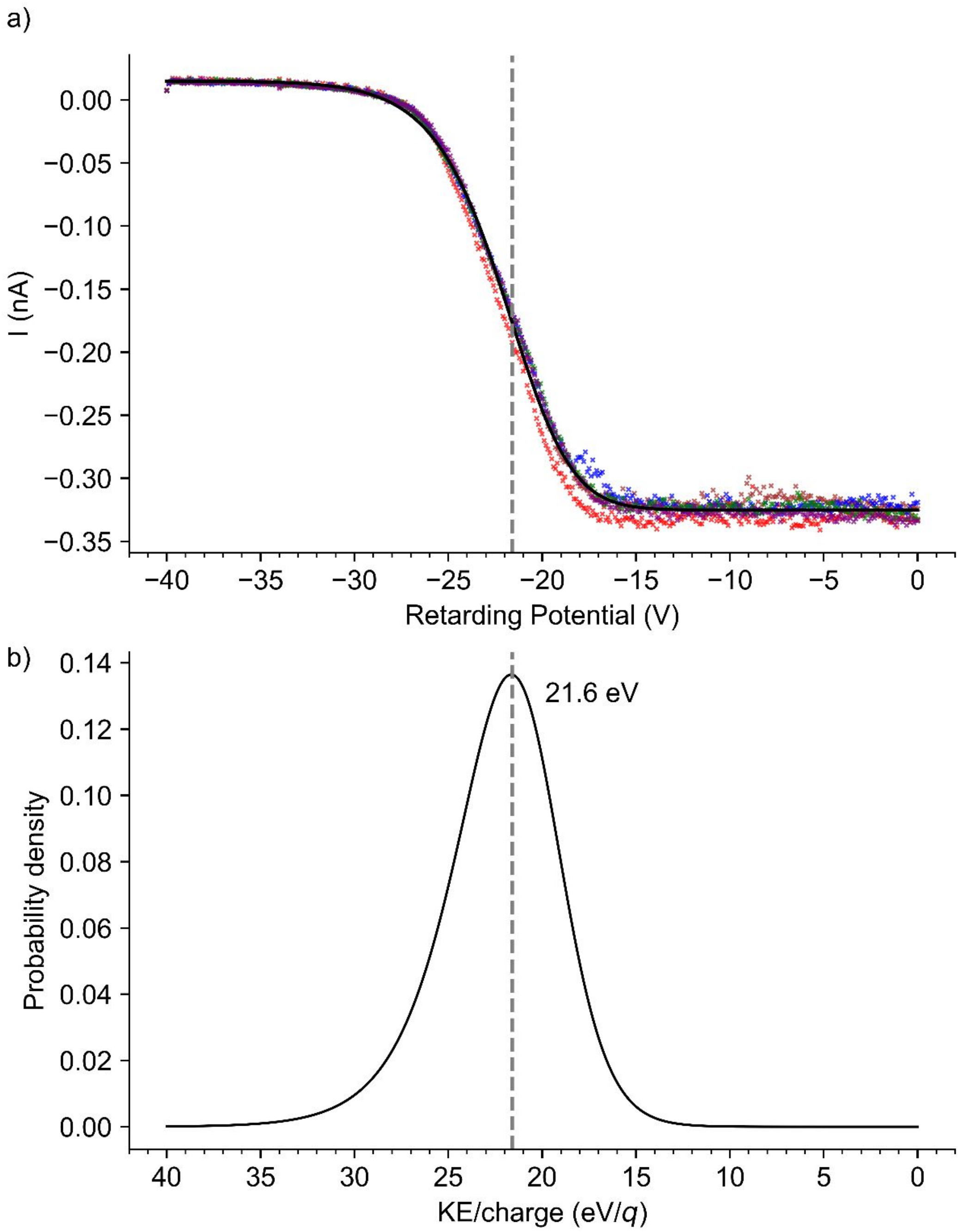

Figure S12. a) Ion current of anion (iii) as a function of the retarding potential. Five separate experiments under identical conditions were performed (individual data points are shown as "X" in a color representative for a single experiment) and all data was subsequently averaged and fitted by a sigmoidal function (black line). The position of the inflection point is marked with a dashed grey line. b) Probability density function for the KE per charge (*q*) obtained by calculating the first derivative of the sigmoidal function in a). The position of the maximum is marked with a dashed grey line and can be understood as most probable KE.

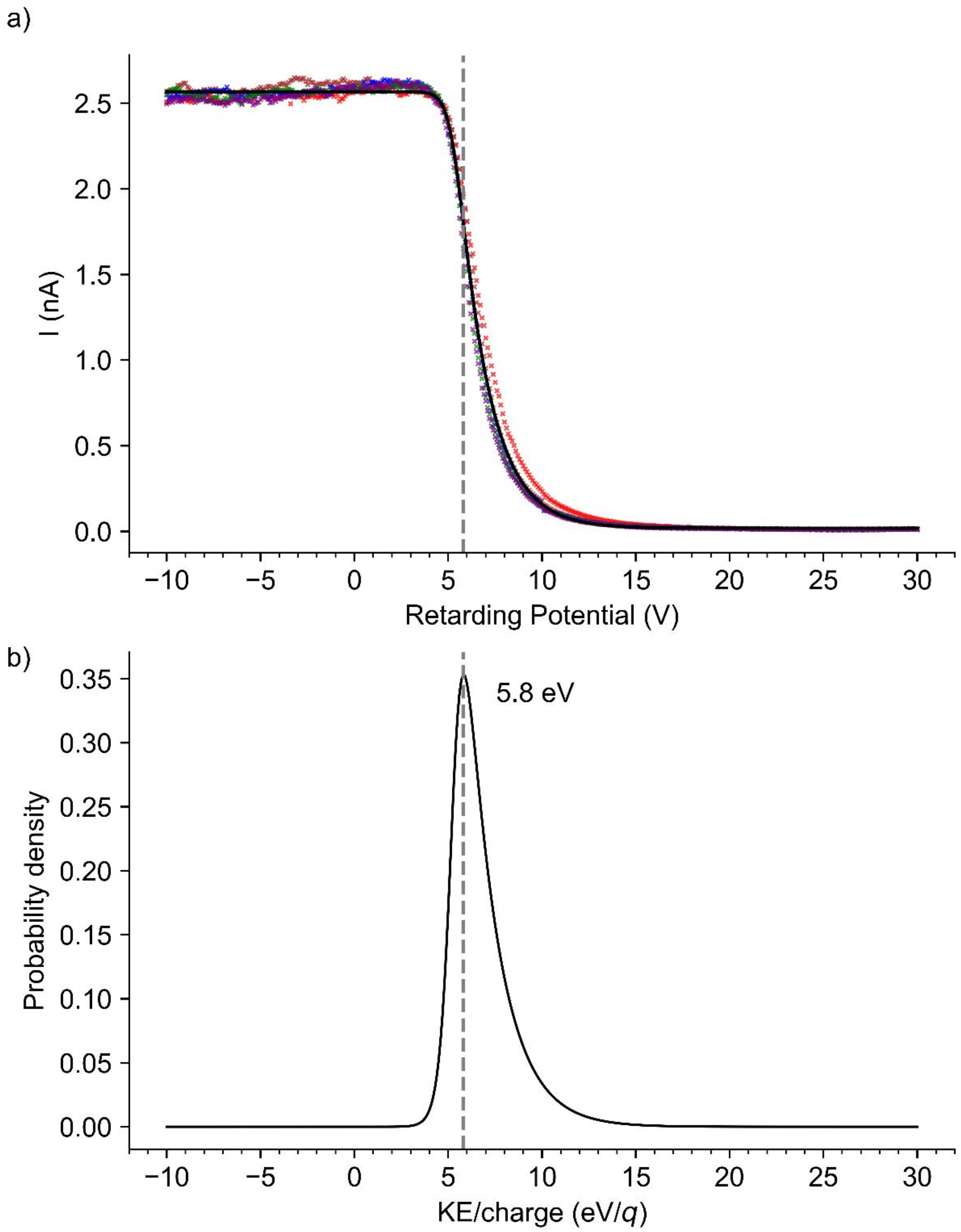

Figure S13. a) Ion current of cation (iv) as a function of the retarding potential. Five separate experiments under identical conditions were performed (individual data points are shown as “X” in a color representative for a single experiment) and all data was subsequently averaged and fitted by a sigmoidal function (black line). The position of the inflection point is marked with a dashed grey line. b) Probability density function for the KE per charge (*q*) obtained by calculating the first derivative of the sigmoidal function in a). The position of the maximum is marked with a dashed grey line and can be understood as most probable KE.

Fragment spectra for gas-phase adducts of $[B_{12}I_{11}]^-$

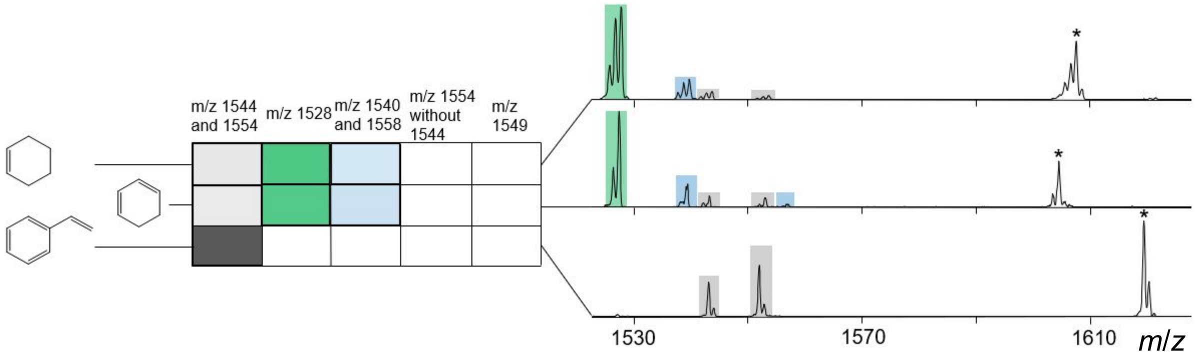

Figure S14. Fragment spectra obtained after isolation and fragmentation of the gas-phase adducts of $[B_{12}I_{11}]^-$ with three selected hydrocarbons. The color code corresponds to the conventions introduced in Figure 1. Remaining adduct signals are marked with asterisks. Note that the types of ions denoted in the table correspond to the respective $[B_{12}Br_{11}]^-$ analogues from Table 1.

Additional fragment spectra for gas-phase adducts of $[B_{12}Br_{11}]^-$

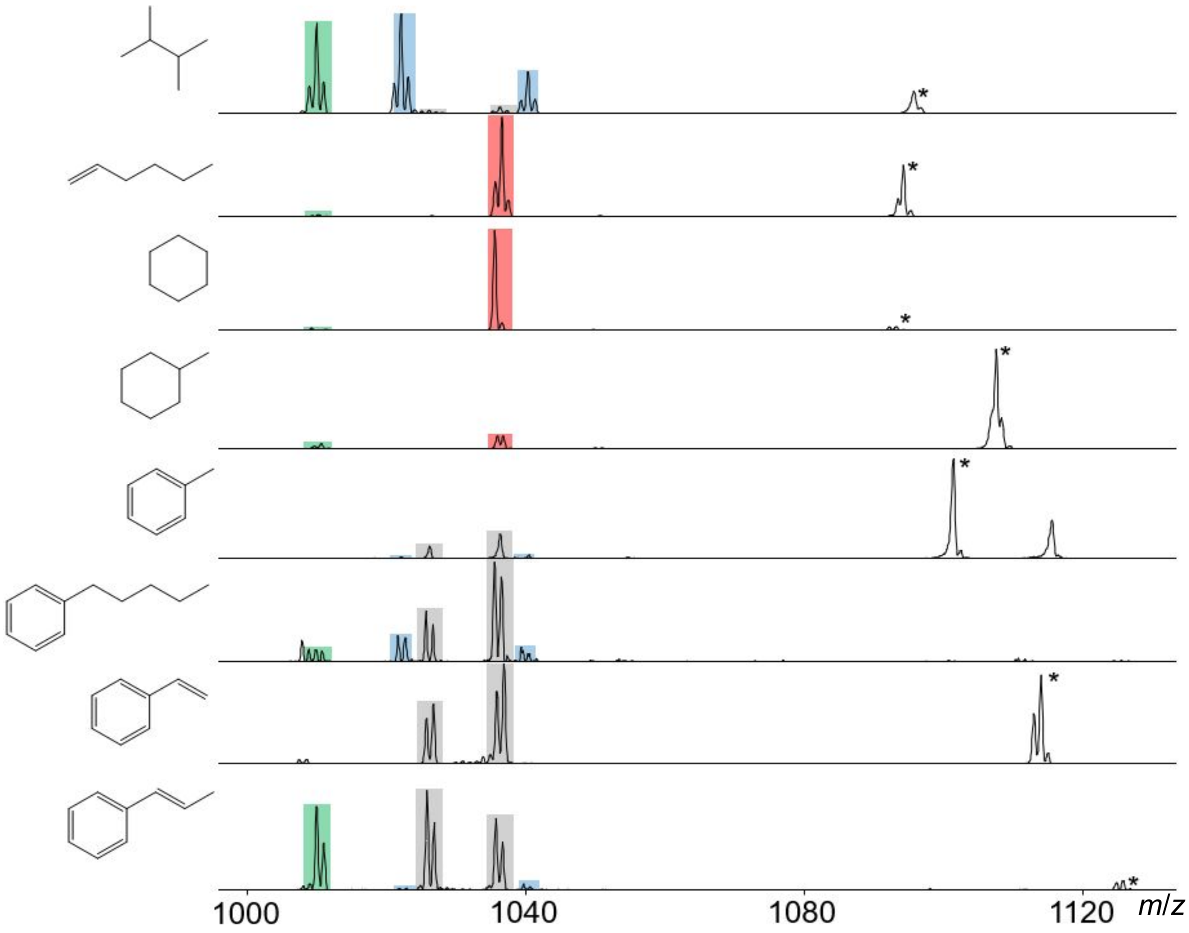

Figure S15. Fragment spectra obtained after isolation and fragmentation of the gas-phase adducts of $[B_{12}Br_{11}]^-$ with the remaining hydrocarbons not explicitly shown in Figure 1. The color code corresponds to the conventions introduced in Figure 1. Remaining adduct signals are marked with asterisks.

Fragment ion deposition with $[B_{12}Br_{11}]^-$

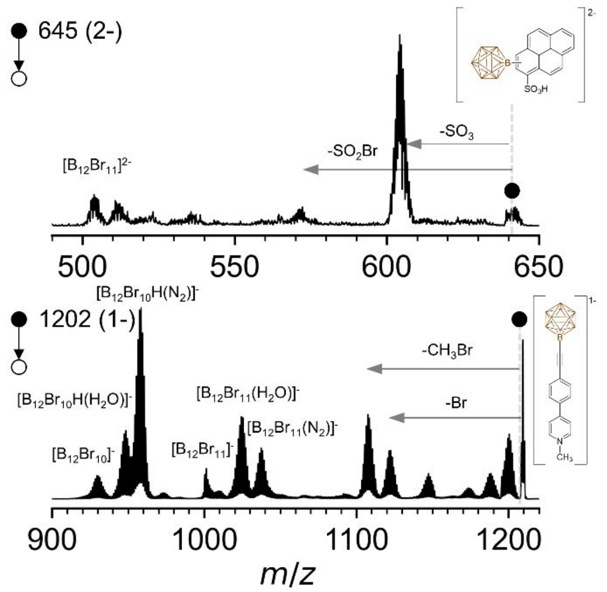

Figure S16. MS² spectrum for the doubly negatively charged adducts of ion (i) with $[B_{12}Br_{11}]^-$ (top), and MS² spectrum for the singly charged adduct of ion (iv) with $[B_{12}Br_{11}]^-$ (bottom). The proposed structure of the main adduct isomer is shown right to the spectra.

Additional MS² spectra for (2-) adducts of ions (i)-(iii) and (1-) adduct of ion (iv)

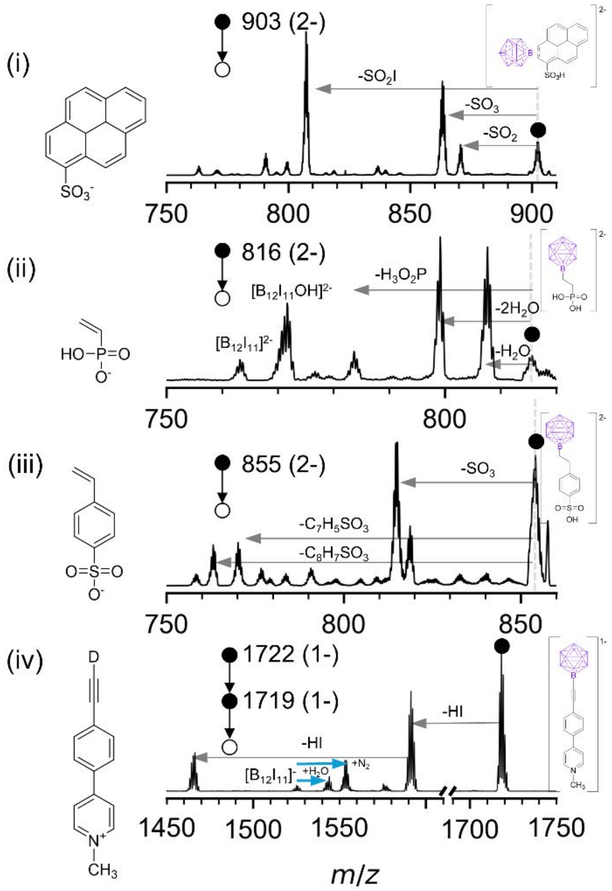

Figure S17. MS² spectra for the doubly negatively charged adducts of ions (i)-(iii) with $[B_{12}I_{11}]^-$, and MS³ spectrum for the singly charged adduct of ion (iv) with $[B_{12}I_{11}]^-$. The proposed structure of the main adduct isomer is shown right to the spectra. The fragmentation reactions can be also found in Scheme 2.

Determination of the D/H ratio in ion (iv)

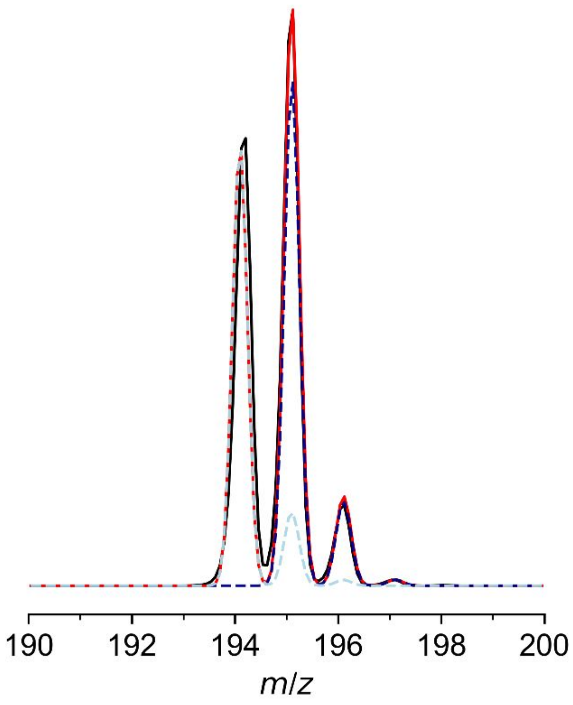

Figure S18. +ESI ion trap MS spectrum acquired from the solution of N-methyl-4-(4-ethynylphenyl-1-yl)pyridinium trifluoromethanesulfonate in ACN that was also used for ion soft-landing. The signal in the region of cation (iv) was fitted by a combination of $[C_{14}H_{11}DN]^+$ (dark blue) and $[C_{14}H_{12}N]^+$ (light blue) to account for the possibility of an exchange of D by H in solution. The overall fit (red) was optimized to best account for the experimental MS spectrum (black) and reveals that at the time of acquisition, the solution contains roughly equal amounts of $[C_{14}H_{11}DN]^+$ and $[C_{14}H_{12}N]^+$. This ratio was then also used to fit the ion pair region in the sequential co-deposition experiment of cation (iv) and $[B_{12}I_{11}]^-$.

Fit of the MS² spectrum of the adduct of ion (iv)

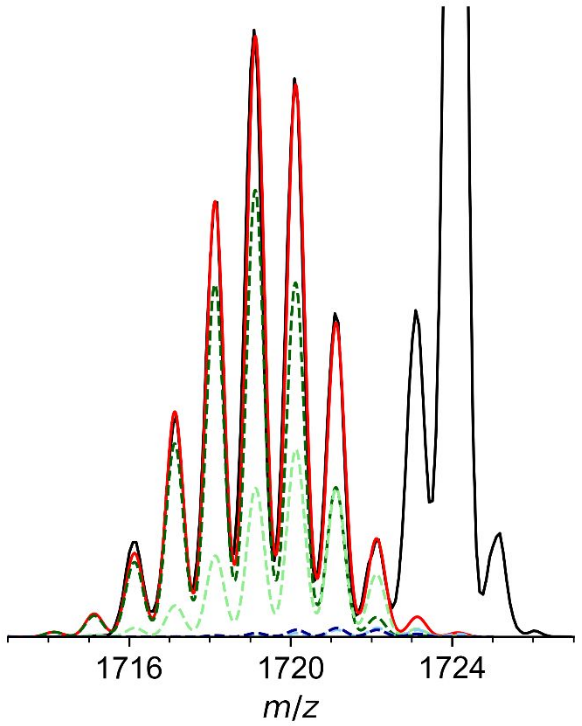

Figure S19.-ESI ion trap MS² spectrum (black) acquired after isolation of the entire isotopic envelope in the region m/z 1715-1727 for the sequential co-deposition experiment of cation (iv) and $[B_{12}I_{11}]^-$. After applying fragmentation energy (CID 25 arb. units), the ion pair intensities involving $[B_{12}I_{11}H]^{2-}$ and cation (iv) vanish while in the adduct region, intensity was preserved. This region was therefore fitted by a combination of D^+ substitution product $[B_{12}I_{11}\text{-}C_{14}H_{11}N]^-$ (69%, dark green), H^+ substitution product (29%, light green) and ion pairs $[[C_{14}H_{11}DN]^+[B_{12}I_{11}H]^{2-}]^-$ (1%, light blue) and $[[C_{14}H_{12}N]^+[B_{12}I_{11}H]^{2-}]^-$ (1%, dark blue) to yield the best overall fit (red) of the experimental MS² spectrum.

Brønsted acidity of selected gas-phase adducts

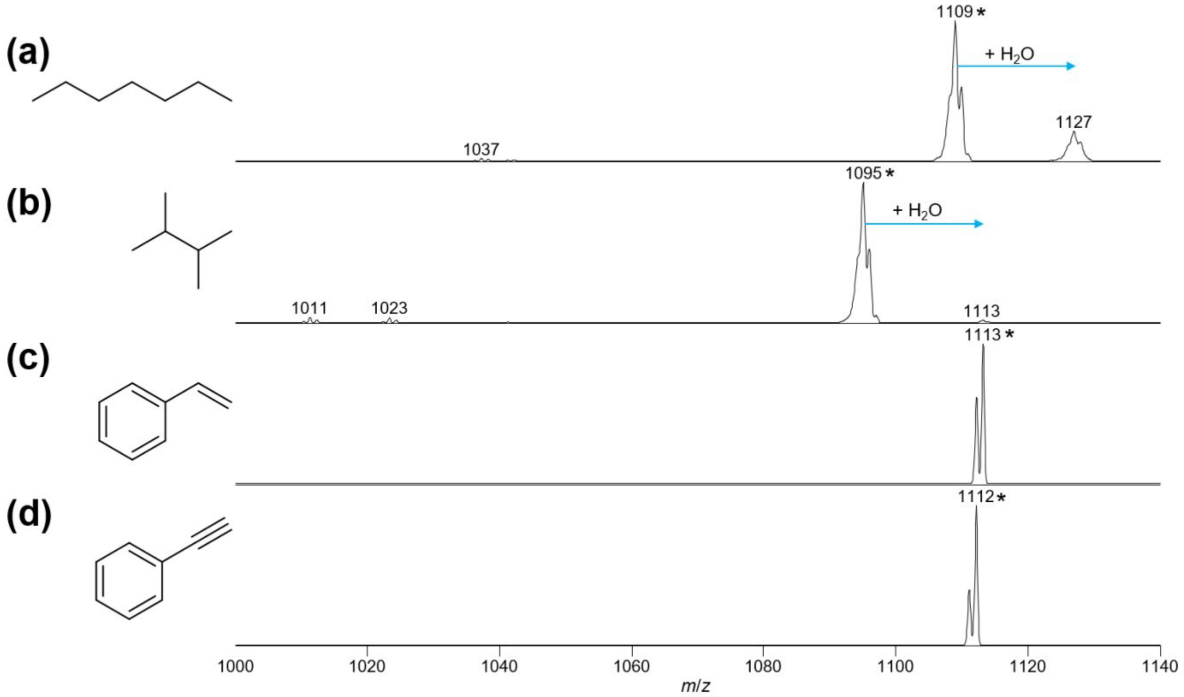

Figure S20. Mass spectra acquired after isolation of the adduct of $[B_{12}Br_{11}]^-$ with (a) *n*-hexane, (b) 2,3-dimethylbutane, (c) styrene, and (d) phenylacetylene. Only in cases (a) and (b), addition of one molecule of H_2O (+18 Da) to the adduct signal was observed, indicating that for these adducts, a free proton is available in the adduct structure for the formation of the ion pair $[H_3O]^+[B_{12}Br_{11}–CH_2–C_nH_{2n+1}]^{2-}$ with residual H_2O in the ion trap. For aromatic compounds (c) and (d), enthalpically more favorable structures than the proton substitution binding mode exist, resulting in the absence of any H_2O addition product due to the absence of sufficient Brønsted acidity of the adduct. The formation of a H_2O addition product is thus indicative of a proton substitution product in the gas phase. Adduct signals are marked with asterisks.

PES diagram for $[B_{12}Br_{11}]^-$ reacting with styrene

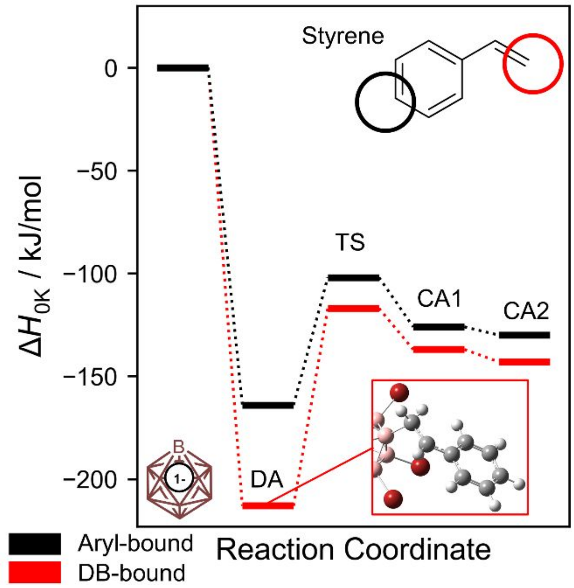

Figure S21. PES diagram (B3LYP-D3BJ/def2-TZVPP) for the reaction of $[B_{12}Br_{11}]^-$ with styrene. The black path shows the reaction of $[B_{12}Br_{11}]^-$ at the aryl site, the red path shows the reaction at the C=C double bond. An extract of the DA minimum geometry is shown in the red frame.

PES diagrams for $[B_{12}I_{11}]^-$ reacting with selected hydrocarbons

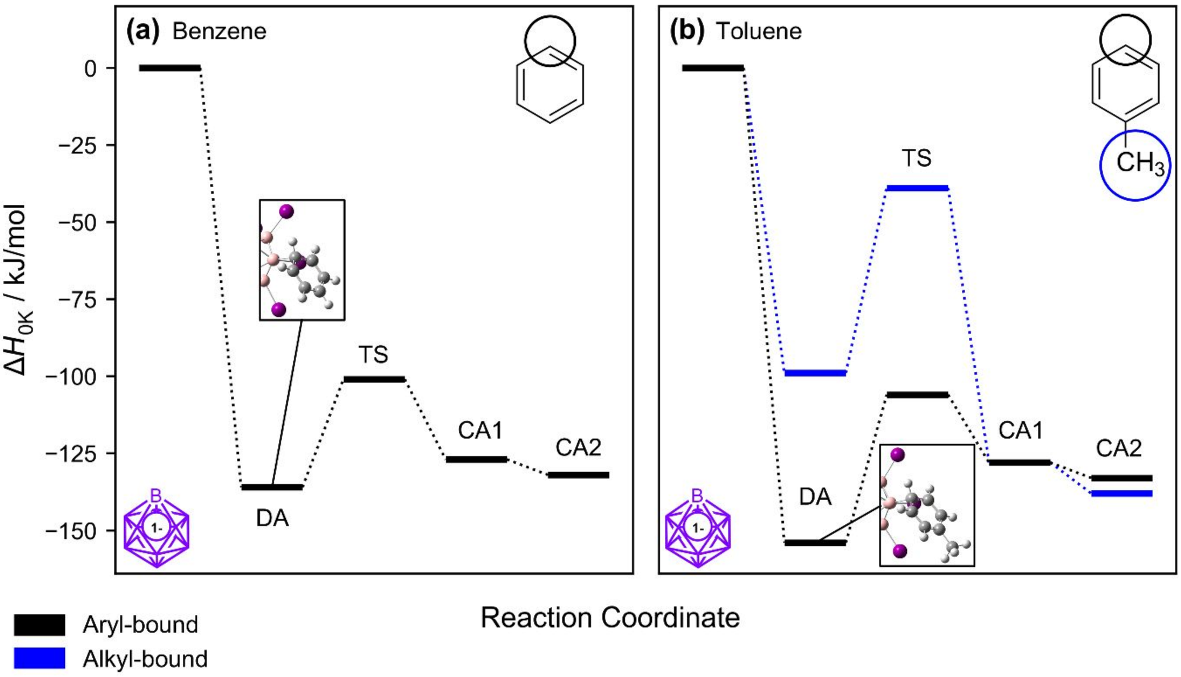

Figure S22. PES diagrams (B3LYP-D3BJ/def2-TZVPP) for the reaction of $[B_{12}I_{11}]^-$ with (a) benzene, and (b) toluene. Black paths show reactions of $[B_{12}I_{11}]^-$ at aryl or olefinic sites of the hydrocarbons, blue paths show reactions at alkyl sites. An extract of the DA minimum geometry is shown in the black frames.

References

(1) Armarego, Wilfred and Chai, C.L.L. *Purification of Laboratory Chemicals*; Elsevier, 2013. https://doi.org/10.1016/C2009-0-64000-9.

(2) Petrovskii, S.; Petrovskaia, A.; Sizova, A.; Sizov, V.; Grachova, E. Homoleptic Alkynylpyridinium Au(I) Complexes as Organometallic 'D-π-A' Chromophores. *ChemPlusChem* **2023**, *88* (7), e202300155. https://doi.org/10.1002/cplu.202300155.

(3) Zhang, Z.; Wu, H.; Xu, W.; Bai, D. Regio- and Diastereoselective Cascade Reactions of Bicyclo[1.1.0]Butanes: Access to *Gem* -Difluorinated Carbocyclic Rings. *Org. Lett.* **2025**, *27* (16), 4378–4383. https://doi.org/10.1021/acs.orglett.5c01132.

(4) Rohdenburg, M.; Warneke, Z.; Knorke, H.; Icker, M.; Warneke, J. Chemical Synthesis with Gaseous Molecular Ions: Harvesting $[B_{12}Br_{11}N_2]^-$ from a Mass Spectrometer. *Angew. Chem. Int. Ed.* **2023**, *62* (45), e202308600. https://doi.org/10.1002/anie.202308600.

(5) Gunaratne, K. D. D.; Prabhakaran, V.; Ibrahim, Y. M.; Norheim, R. V.; Johnson, G. E.; Laskin, J. Design and Performance of a High-Flux Electrospray Ionization Source for Ion Soft Landing. *Analyst* **2015**, *140* (9), 2957–2963. https://doi.org/10.1039/C5AN00220F.

(6) Kawa, S.; Behrend, K. A.; Knorke, H.; Rohdenburg, M.; Volke, D.; Rothemund, S.; Warneke, J. Selective Functionalization of Peptides with Reactive Fragment Ions. *J. Am. Soc. Mass Spectrom.* **2025**, *36* (8), 1779–1790. https://doi.org/10.1021/jasms.5c00145.

(7) Su, P.; Hu, H.; Warneke, J.; Belov, M. E.; Anderson, G. A.; Laskin, J. Design and Performance of a Dual-Polarity Instrument for Ion Soft Landing. *Anal. Chem.* **2019**, *91* (9), 5904–5912. https://doi.org/10.1021/acs.analchem.9b00309.

(8) Rohdenburg, M.; Kawa, S.; Ha-Shan, M.; Reichelt, M.; Knorke, H.; Denecke, R.; Warneke, J. Probing Fragment Ion Reactivity towards Functional Groups on Coordination Polymer Surfaces. *Chem. Commun.* **2024**, *60* (75), 10306–10309. https://doi.org/10.1039/D4CC00767K.
